# ISsaga is an ensemble of web-based methods for high throughput identification and semi-automatic annotation of insertion sequences in prokaryotic genomes

**DOI:** 10.1186/gb-2011-12-3-r30

**Published:** 2011-03-28

**Authors:** Alessandro M Varani, Patricia Siguier, Edith Gourbeyre, Vincent Charneau, Mick Chandler

**Affiliations:** 1 Laboratoire de Microbiologie et Génétique Moléculaires, CNRS 118, Route de Narbonne, 31062 Toulouse Cedex, France

## Abstract

Insertion sequences (ISs) play a key role in prokaryotic genome evolution but are seldom well annotated. We describe a web application pipeline, ISsaga (http://issaga.biotoul.fr/ISsaga/issaga_index.php), that provides computational tools and methods for high-quality IS annotation. It uses established ISfinder annotation standards and permits rapid processing of single or multiple prokaryote genomes. ISsaga provides general prediction and annotation tools, information on genome context of individual ISs and a graphical overview of IS distribution around the genome of interest.

## Background

The growing number of completely sequenced bacterial and archaeal genomes are making important contributions to understanding genome structure and evolution. Annotation of gene content and genome comparison have also provided much valuable information and key insights into how prokaryotes are genetically tailored to their lifestyles.

The rate at which sequenced prokaryotic genomes and metagenomes are accumulating is constantly increasing with the development of new high-throughput sequencing techniques. The resulting mass of data should provide an unparalleled opportunity to achieve a better understanding of prokaryotes. High quality genome annotation together with a standardized nomenclature is an essential requirement for this since most proteins identified from these sequencing projects will probably never be characterized biochemically [1]. Unfortunately, expert genome annotation is fast becoming a bottleneck in genomics [2].

A crucial example of an annotation bottleneck concerns insertion sequences (ISs), the smallest and simplest autonomous mobile genetic elements. These contribute massively to horizontal gene transfer and play a key role in genome organization and evolution, but are seldom correctly annotated at the DNA level. ISs are transposable DNA segments ranging from 0.7 to 3.5 kbp, generally including a transposase gene encoding the enzyme that catalyses IS movement. Many (but not all) ISs are delimited by short terminal inverted repeat (IR) sequences and flanked by short, direct repeat (DR) sequences. The DRs are generated in the target DNA as a result of insertion. ISs are classified into about 25 different families on the basis of the relatedness of transposases and overall organization (ISfinder) [3]. They are often present in significant numbers in prokaryote genomes and, indeed, transposases are by far the most abundant and ubiquitous genes found in nature [4].

Available annotation programs do not provide an authoritative IS annotation. Correct annotation must include both protein and DNA. These features are characteristic for each IS family and provide information concerning their mechanism of transposition and their possible roles in modifying the host genome. At the protein level, transposases are often mislabeled as 'integrase', 'recombinase', 'protein of unknown function' or 'hypothetical protein'. Moreover, IS-associated accessory (often regulatory) and other passenger genes are rarely correctly described. At the DNA level, features such as the IRs and DRs, whose presence can indicate whether the IS is potentially active, are generally missing. Partial IS copies are even more rarely annotated. Partial IS copies are important because they represent scars of ancestral recombination events and, as such, can provide information concerning the evolution of the host replicon.

Additional IS-related genetic objects, such as miniature inverted repeat transposable elements (MITEs), mobile insertion cassettes (MICs) and solo IRs [5], are also missing from the majority of genome annotations. Some of these structures, although not encoding their own transposase, can be activated by a cognate transposase from an intact related IS also present in the genome and therefore can impact on genome evolution. More recently, IS copies including additional passenger genes unrelated to transposition (transporter ISs) have been identified, confounding the frontier between ISs and transposons [6]. Although ISs are relatively simple genetic objects, they are sufficiently diverse in sequence and organization that their annotation is not simple and presents some major hurdles for automatic annotation systems. The failure to accurately annotate ISs in publicly available prokaryote genomes severely biases studies attempting to provide an overview of IS distributions related to prokaryotic phylogenies or ecological niches.

To overcome the present annotation limitations, we have developed ISsaga (Insertion Sequence semi-automatic genome annotation), which provides comprehensive computational tools and methods for rapid, high-quality IS annotation. This is integrated as a module into ISfinder, the prokaryote IS reference centre database [7] and IS repository, which includes more than 3,500 expertly annotated individual ISs from bacteria and archaea and also provides a basis for IS classification. ISsaga is part of the ISfinder 'Genome' section, which also includes ISbrowser, a genome visualization tool for ISs, which at present contains more than 40 expertly annotated genomes (119 replicons). The ISsaga platform has been designed to maintain common standards for high quality IS annotation used in ISfinder at both protein and nucleotide levels. It is a web-based service that includes an ensemble of methods for IS identification and is freely available to the academic community.

We have successfully tested this new software suite using several genomes available in the public databases and find that it provides a significantly more complete picture of each of these genomes than is presently available. The annotation quality obtained with ISsaga approached that which ISfinder experts obtain with our manual methods [6].

## Results

### ISsaga overview

#### What is ISsaga?

**Figure 1:**
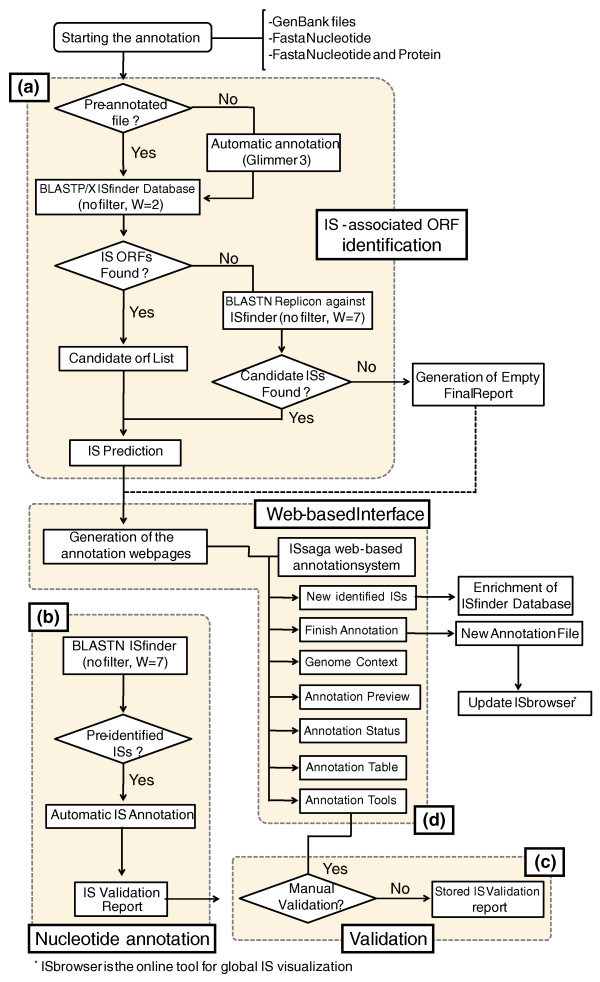
**Flow diagram of the ISsaga pipeline**. The figure shows how the different ISsaga functions are assembled. Following loading of the appropriate genome file, the system identifies ORFs using the ORF identification module. Module **(a)**: if the file is pre-annotated, the protocol performs a BLASTP (filter off and e-value 1e-5) analysis followed by BLASTX (filter off and e-value 1e-5) to identify any ORFs that may have been overlooked. If the file is not annotated, an automatic Glimmer annotation is performed prior to BLASTP and BLASTX. Identified ORFs are included in a candidate ORF list. The replicon is then subject to BLASTN (filter off, word size 7 and e-value 1e-5) analysis, which yields an IS prediction and generates a web-based annotation table. If no ORFs are found, BLASTN is performed against the ISfinder database and any candidate ISs are fed into the IS prediction step. This step identifies partial ISs without ORFs. In a second module **(b)**, ISs that have been identified and are already present in ISfinder are automatically fed into an IS report that must then be validated (module **(c)**). These modules are linked to the web interface (module **(d)**), which permits annotation management and provides tools for identifying and defining new ISs.

ISsaga is designed specifically for use with the ISfinder database and leads the annotator simply through the annotation process in a sequential manner. A flow chart describing the system is shown in Figure 1. The annotation process requires a user quality control, which is described in the ISsaga manual (Additional file 1) or can be supplied by expert ISfinder annotators on request. ISsaga is a semi-automatic system in which all automatically generated results must be validated by the user. The user must also identify any new IS elements not already present in ISfinder using the toolbox provided by the system. These procedures are explained in detail in the user manual.

Although the system is provided freely to the academic community, its use requires registration. This step protects the data of individual users and ensures that correct annotation standards are used. The fact that transposases are the most ubiquitous genes found in nature [4], together with the number of incorrectly annotated genomes we have encountered in the public databases (in which errors are often widely propagated and difficult to correct *a posteriori*), makes this constraint essential. In opening an annotation project in ISsaga, the user has the choice of retaining the final annotations in a private section (where they will be retained for 6 months before transfer to ISfinder and ISbrowser) or including it directly in the public databases. Note that each addition to ISfinder increases the efficiency of annotation of subsequent genomes and the database therefore depends on contributions from the community.

The semi-automatic annotation system uses the Blast [8] algorithm in two modules: protein and nucleotide annotation. Each module consists of a group of programs written in BioPerl [9], Bourne Shell and PHP languages and executed in the http Apache manager (version 2.2.12), together with a database implemented by MySQL (version 5.1.37).

Examples of a completed genome annotation and a genome 'in progress' performed using ISsaga can be found on the web site without registration. Selected tabs that are important for understanding the description below are indicated in the accompanying text in the form: (Tab/'Link'). A complete manual can also be consulted online or downloaded as a '.pdf' file (see also Additional file 1).

#### Genome file format and loading

ISsaga accepts pre-annotated GenBank files (.gbk), the recommended format, and FASTA nucleotide files (.fasta). It will also accept FASTA protein files (.faa) but only together with the corresponding FASTA nucleotide file. It performs automatic IS-associated ORF identification using IS-associated transposase and transposition-related (for example, regulatory) gene models (provided by ISfinder) for '.fasta' input files. The recommended genome input file for ISsaga is the GenBank format because this file format normally includes pseudogene annotations. The system can be used to annotate ten replicons concurrently in a single project (that is, including several chromosomes and plasmids that may constitute the genome of interest).

#### IS-associated ORF identification

The first step in the ISsaga pipeline is identification of IS-associated ORFs. This is performed by the ORF identification module (module (a) in Figure 1), which identifies IS-associated ORFs within a given genome and attributes them to IS families defined in ISfinder.

With a single genomic nucleotide FASTA file (.fasta) the platform will automatically predict all IS-associated ORFs using Glimmer3 [10] with an optimized gene model derived from the ISfinder dataset. If provided with the corresponding '.faa' file, the system will consider this as an annotated file and will not perform the initial ORF identification step.

To verify that all ORFs of potential interest have been identified, a BLASTX analysis is then performed. A web-based interface will show the predicted number of ISs and families and distinguish partial from full copies. This serves simply as a guide to aid the user through the nucleotide and validation modules. An annotation table (Annotation tab/'Annotation Table') is also generated (Additional file 2). This will be gradually completed during the annotation process. It includes the ORFs identified, their family attribution, and similarity with ISs in ISfinder as well as their genome coordinates. It also contains fields concerning the subsequent nucleotide annotation (Additional file 2).

If a member of a new family exists and its transposase has been annotated as such in the source GenBank file, ISsaga will provide it with a tag 'putative new family'. Clearly, ISsaga will not automatically identify ISs that are very different to those in the database and whose transposases have not been previously annotated. For example, those ISs that transpose by different chemistries to the classical aspartate-aspartate-glutamate catalytic domain (DDE) transposases will not be found unless a copy is included in ISfinder. Contributions from the community obtained from direct identification of ISs from individual transposition events (for example, insertional mutation of cloned genes) is important in improving IS identification and extending the accuracy of annotation. The probability of not identifying ISs will decrease with the increasing use of ISsaga to supplement the ISfinder database.

#### IS nucleotide sequence annotation

The nucleotide annotation module (module (b) in Figure 1) automatically identifies ISs already present in ISfinder. It generates a list of ISs present in the genome (Semi-automatic tab/'List Annotated IS(s)') and a report for each IS, including details of each individual copy. These must be validated by the user and will then be automatically added to the annotation table.

If an ORF does not correspond to the transposase of an IS present in ISfinder, the corresponding IS must be defined by the user. This will be the reference IS, which will be added to ISfinder. ISsaga includes a tool box (Tools tab) with a detailed explanation for this purpose. Once the program has estimated the number of new ISs, ISfinder will, on request, attribute a block of names (one for each new IS) using the standard nomenclature system. The user should submit the new ISs to ISfinder for verification using the direct IS submission tool (Validation tab/'Submit IS to ISfinder'). These will then be included automatically in ISfinder (either in the public or private sections, as initially chosen by the user when opening the project). The new ISs will be added to the list of ISs present in the genome and a report generated, which, after validation, will be added to the annotation table (Additional file 2).

Prokaryotic genomes often carry intercalated IS clusters in which one IS is interrupted by insertion of additional ISs. ISsaga includes a tool in the annotation report to resolve such structures and to reconstruct the associated ISs.

#### Following annotation progress

During the annotation process the user can generate a series of graphic representations of the annotation status (Annotation tab/'Annotation Status'), including a pie chart and histograms as well as a circular representation of the IS distribution using an integrated CGView tool [11] (Annotation tab/'ISbrowser Preview') This is only accessible from a 'replicon page', not from the 'project page' (see manual). This feature, integrated into ISbrowser [12], is dynamic and, together with a summary table, provides a continuous snapshot of progress of the annotation. This can be compared directly with the results obtained from the automatic prediction (Annotation tab/'Global Annotation Prediction').

#### ISsaga output

**Figure 2:**
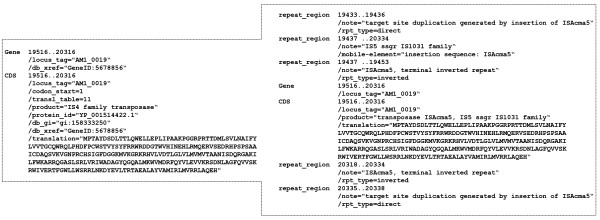
**A section of the original GenBank file (left) and of the extracted file after correct annotation using ISsaga**.

At the end of the annotation process (when all lines in the annotation table are complete), the identified IS(s) and the annotation result can be retrieved in a spread-sheet format or as a new GenBank file (Annotation tab/'Extract Annotation'). The possibility of extracting a new and correct GenBank file (Figure 2) will facilitate replacement of partial or badly annotated files and reduce subsequent propagation of errors to other genomes. The corrected file can be exported to applications such as Artemis [13] and Gbrowser [14] for further analysis.

It will also be possible, in the near future, to export the results to ISbrowser. For this, the completed annotation must first be validated and curated by ISfinder.

### Testing ISsaga reliability

#### Rapid estimation of IS content

In many cases, a user does not necessarily need an accurate annotation but would simply like to obtain an estimate of the number of ISs (both complete and partial copies) and the number of different IS families in a given genome. This can be obtained using Annotation tab/'Replicon Annotation Prediction'. The prediction is automatically generated in the initial step after loading the genome file. We have introduced a number of rules that operate automatically to remove many of the major annotation ambiguities encountered due to the diversity and complexity of ISs (for example, the presence of more than one ORF in an IS, overlapping reading frames, programmed translational frameshifting, and so on). These rules are not exhaustive. They have been defined from our present experience with IS identification but, as more such cases come to light, additional rules will be added.

#### Comparison of ISsaga prediction with available annotated genomes

We have tested the ISsaga prediction tool using eight bacterial chromosomes chosen to represent different types of IS population, including high and low IS density, intercalated clusters of ISs and a wide variety of IS families (both as complete and partial copies). We compared the results obtained with the prediction tool, those obtained by expert annotation through the standard ISfinder procedure as described by Siguier *et al*. [6] and the original annotated GenBank files. The genomes analysed were *Clostridium thermocellum*, two strains of *Stenotrophomonas maltophilia*, two strains of *Anaeromyxobacter *sp., two strains of *Anaeromyxobacter dehalogenans *and *Aquiflex aeolicus *(Table 1). Clearly, the annotations included in the original GenBank file severely underestimate both the number and diversity of the IS population in each of the chosen genomes compared with those identified using manual ISfinder annotation. Where annotations exist in the GenBank files, these generally only concern proteins that carry a tag 'transposase' with no indication of IS family. If an IS family is attributed, it is often incorrect (for example, 'mutator', a eukaryote transposon, instead of the prokaryotic IS*256*, or IS*4*, which is attributed to a large proportion of classical transposases). In addition, it is even more common that no nucleotide annotation is included.

**Table 1:** Predictor performance

	GB	- IS	+ IS	Manual
***A. dehalogenans *2CPC (NC_007760)**				
Total IS ORF	1	4	4	2
Complete ORF	-	0	0	0
Partial ORF	-	1	1	1
Pseudogene	1	2	2	1
Unknown ORF	-	1	1	0
Total IS	-	4	4	2
Different IS	-	4	4	2
				
***Anaeromyxobacter *sp. Fw109 5 (NC_009675)**				
Total IS ORF	15	22	24	19
Complete ORF	-	4	12	12
Partial ORF	-	1	2	6
Pseudogene	1	4	4	1
Unknown ORF	-	13	6	0
Total IS	-	20	21	16
Different IS	-	16	17	12
				
***Anaeromyxobacter *sp. K (NC_011145)**				
Total IS ORF	14	25	28	27
Complete ORF	-	12	26	26
Partial ORF	-	2	0	0
Pseudogene	-	1	1	1
Unknown ORF	-	10	1	0
Total IS	-	19	19	18
Different IS	-	10	10	9
				
***A. dehalogenans *2CP1 (NC_011891)**				
Total IS ORF	15	33	35	35
Complete ORF	-	18	24	27
Partial ORF	-	4	2	3
Pseudogene	-	8	8	5
Unknown ORF	-	3	1	0
Total IS	-	25	25	23
Different IS	-	12	12	14
				
***A. aeolicus *VF5 (NC_000918)**				
Total IS ORF	-	7	7	3
Complete ORF	-	0	2	2
Partial ORF	-	1	1	1
Pseudogene	-	0	0	0
Unknown ORF	-	6	4	0
Total IS	-	7	7	3
Different IS	-	6	6	2
				
***C. thermocellum *27405 (NC_009012)**				
Total IS ORF	75	143	144	160
Complete ORF	-	81	123	125
Partial ORF	-	43	11	27
Pseudogene	-	7	7	8
Unknown ORF	-	12	3	0
Total IS	-	115	115	119
Different IS	-	27	27	26
				
***S. maltophilia *R5513 (NC_011071)**				
Total IS ORF	11	21	22	20
Complete ORF	-	13	19	19
Partial ORF	-	7	1	1
Pseudogene	-	1	1	0
Unknown ORF	-	0	1	0
Total IS	-	18	19	16
Different IS	-	6	7	4
				
***S. maltophilia *K279a (NC_010943)**				
Total IS ORF	49	53	54	57
Complete ORF	-	18	45	47
Partial ORF	-	27	5	9
Pseudogene	-	3	3	1
Unknown ORF	3	5	1	0
Total IS	-	38	39	36
Different IS	-	18	19	18

The table shows a comparison of IS annotations of eight bacterial genomes contained in the corresponding GenBank files (GB) with those obtained by manual annotation (Manual) and using the ISsaga predictor with two different IS reference databases. In one database (-IS) the reference ISs contained in the genome under test were removed while in the other these ISs were included (+IS). The total number of IS-associated ORFs (Total IS ORF) are divided into four categories: Complete ORFs, Partial ORFs, Pseudogenes and Unknown. The category 'Unknown' includes all examples that cannot be distinguished by the predictor as complete or partial due to the absence of sufficient numbers of closely related examples in the reference database. The categories 'Total IS' and 'Different IS' are based on nucleotide predictions. In these predictions the number of ORFs carried by the IS are taken into account. For example, if an IS includes two ORFs, this will be counted as two examples in 'Complete ORF' but as a single IS in 'Total IS'.

The number of predictor-identified ORFs approaches that obtained by manual ISfinder annotation [6]. In certain cases, however, the predictor provides an overestimate. When investigated individually, these were found to be of two major types. The first class includes proteins similar to accessory proteins of the IS*91 *and Tn*3 *families, such as tyrosine or serine recombinases (integrases and resolvases, respectively). The second class contains proteins that share a domain with an accessory IS gene (that is, not a transposase), for example, the ATP binding domain of the IS*21 *'helper' protein, IstB. Although we have included filters to eliminate some of these, we have voluntarily set the filters at a level that retains a small fraction. This ensures that we do not eliminate real but distantly related IS-associated ORFs. Another reason for over-estimating the total number of ISs is that ISsaga will consider an interrupted IS ORF (relatively frequent events) as two or more occurrences. We cannot supply filters for these unless the IS is included in ISfinder, and the user must reconstruct the sequence manually.

Although many false positives are removed from the predictor results, they are included in the final annotation table. This permits individual examination and manual deletion or validation in the final annotation.

In spite of the limitations of the predictor, we emphasize that it remains the most reliable available software for automatic IS prediction and its reliability will evolve with time and experience.

### Exploitation of ISsaga

#### Genome context

**Figure 3:**
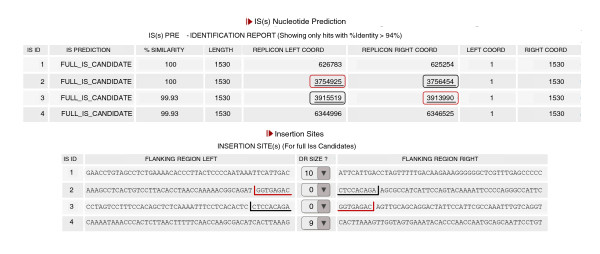
**Part of the individual IS report**. This example shows the four complete copies of IS*Acma18 *from the genome of *Acaryochloris marina*. The top section shows the genome coordinates of each IS. Note that copies 2 and 3 are at some distance from each other. The lower section shows the flanking 49 bp and the corresponding DRs. Note that the left 'DR' of copy 2 (marked in red) is present as the right 'DR' of copy 3 (marked in red) whereas the right 'DR' of copy 2 (marked in black) is present as the left 'DR' of copy 3 (marked in black).

One useful feature of ISsaga is that it supplies the genome context (that is, flanking genes) for each annotated IS, allowing identification of IS-induced gene disruption and rearrangements. For example, the DRs flanking an IS are generated by insertion into a specific site. If a particular IS does not exhibit flanking DRs but other ISs of the same family do, it is likely that this IS has been involved in a rearrangement either by transposition or by homologous recombination with a second copy. The individual IS report (Semi-automatic tab/'List Annotated IS(s)') (Figure 3) presents a list of IS target sites together with the flanking regions, including DRs (when present). Inspection of this can often reveal the presence of one DR copy associated with one IS while the other is associated with a second IS in the list. This indicates where recombination has occurred or, alternatively, the point of insertion of a composite transposon (in which a segment of DNA is flanked by two similar ISs in direct or inverted relative orientation). In the example given, the distance between the two ISs concerned is too great for a composite transposon, implying that an IS-mediated rearrangement has occurred. It is also possible that the analysis will provide evidence of IS-mediated synteny interruption between two closely related strains (for example, [15]).

Additionally, inspection of flanking genes or gene fragments can uncover a variety of local genomic modifications: genes interrupted by the insertion; insertional hotspots relating to target specificity; intercalated or tandem ISs; and IS-driven flanking gene expression (for example, formation of hybrid promoters) [3].

The ability to identify partial IS copies, intercalated ISs and IS derivatives, such as MITEs, MICs, and solo IRs, as well as more complex structures, such as ISs with passenger genes and new potential compound transposons, is important. Their inclusion gives a significantly more accurate interpretation of the spread and distribution of ISs and provides information about the evolutionary history of the host genome. This topic periodically receives attention but, since the analyses are generally based on extremely limited, incomplete and inaccurate data sets, most of the published results have very limited utility.

## Discussion

Machine-based genome annotation, when coupled to an expertly curated reference database, represents a powerful combination for providing high quality data, especially when subject to expert human inspection and validation. The numerical importance of transposases in nature [4], and presumably, therefore, the genetic objects on which they function, makes their correct annotation imperative. However, although ISs are arguably the simplest autonomous transposable elements, their diversity and complexity probably exclude the development of an entirely automatic annotation procedure. While ISsaga is only semi-automatic and requires some user input and expertise, it permits accurate and relatively rapid IS annotation. Moreover, as the ISfinder database is enriched, the automatic step of IS identification and annotation will steadily improve by reducing the user input and the time necessary to define uncharacterized ISs in the genome.

### Genome assembly

ISsaga can also assist genome assembly in sequencing projects. Complete genome sequencing involves assembly of 'contigs' into a complete replicon. Due to the limitations of assembly programs, the presence of repeated sequences such as ISs, often located at the contig ends, complicates the assembly procedure. A knowledge of IS context resulting from accurate annotation of individual contigs can assist in genome assembly.

The increased sequencing capacities now available have also led to a more pragmatic approach for rapid comparison of sets of closely related strains in which contigs are simply mapped to a common scaffold rather than assembled into a definitive genome [16]. Again, since many contigs are terminated by repeated sequences, IS context obtained from accurate annotation can provide strong support for assembly of the scaffold for synteny studies.

### Metagenomes

Increased sequencing capacity has also resulted in a paradigm shift from genome-centric to gene-centric approaches with the advent of metagenomics. ISsaga can contribute fundamentally to such studies in two ways: firstly by enriching the ISfinder database by high throughput annotation of completely assembled and scaffold-based genomes; and secondly by direct analysis of the metagenomes themselves. Although typical sequence runs in metagenomic analyses are short, enough information can be present to identify a particular IS from fragments at the DNA or protein level. Again, IS context provided by ISsaga could assist in small assemblies but, more importantly, it will provide identification tags for ISs whose distribution is limited and that may be used to determine some of the genera and even species present in the original sample.

### Genome evolution

Another advantage provided by a complete genome IS annotation is that it permits a detailed basis on which to compare strains and species. An excellent example is that of the Bordetellae [17], in which IS activity has had a profound effect on the structure and size of several different species in a process that can be correlated with pathogenicity.

### Other mobile genetic elements

ISs and IS derivatives represent only a proportion of all prokaryotic mobile genetic elements. It is hoped that ISsaga will be extended to other mobile genetic elements such as transposons, integrative conjugative elements (ICEs) [18] and integrons [19].

It is expected that the ISsaga pipeline and its future development will provide the scientific community with a significantly more accurate way of annotating their own set of this type of mobile genetic element and in sharing the expertise of ISfinder through the web service.

## Materials and methods

### ISfinder annotation procedure as used in ISsaga

ISsaga uses a semi-automatic procedure based on the methodology for identification of ISs in the public databases described in [6].

ISsaga has a semi-automatic and manual modular architecture described in detail in Figure 1, in the user manual (Additional file 1 and [20]) and largely in the body of this article. The modular construction allows the annotation process to be broken down into three interconnected steps: protein (IS-associated ORF identification); nucleotide; and validation steps.

For the web interface ISsaga uses PHP [21] in the http Apache manager (version 2.2.12). The execution procedure in each annotation module was written in BioPerl [9] and Bourne Shell languages and executed with a database implemented by MySQL (version 5.1.37). Both use a set of open source software described in the user manual.

The protein and nucleotide steps are entirely based on sequence similarity comparison using BLAST [8] software against a daily updated version of the ISfinder database. The protein step, includes determination of the IS-associated (complete/intact or partial/fragment) genes and the transposase family, optimized by the BlastP and BlastX parameters (similarity threshold of more than 97%, word size of 3, e-value 1e-5 and the complexity filter disabled). ISsaga scans the input genome annotation for IS-associated ORFs. All ORFs inside the blast threshold are considered as potential IS regions.

For unannotated genomes (fasta file input), a prior ORF prediction is automatically made with Glimmer3 using a specific IS-associated gene model constructed with the 'build-icm' program (provided by the Glimmer3 package) with the training set provided by the ISfinder protein sequence database. The results of this step are included in the annotation table (Additional file 2).

**Figure 4:**
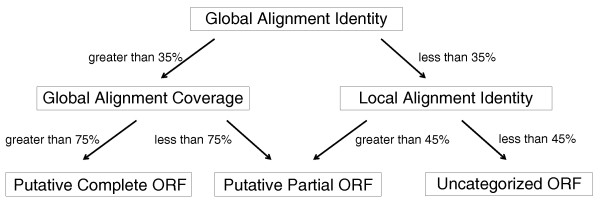
**Decision tree to determine complete, partial or uncategorized IS-associated ORFs based in global and local alignments against the ISfinder protein dataset**.

The IS ORF prediction (complete, partial or uncategorized) uses both global (Emboss stretcher) and local (Blast) alignment procedures against the ISfinder protein dataset (Figure 4).

For IS nucleotide prediction, ISsaga takes into account the characteristics of each IS family (as defined on the ISfinder website) to identify the regions that could contain an IS. For example, for an IS composed of two ORFs, ISsaga will extract the nucleotide sequence starting from the coordinates of the beginning of the first ORF to the coordinates of the end of the second. All nucleotide candidate IS regions are grouped by Blastclust program (parameters: -p F -S 90 -b F -L 0.0) to determine the number of different regions.

The nucleotide step includes identification of the IRs or IS ends, and the insertion site with DRs of each IS-associated ORF previously identified, and for putative partial ISs that do not contain ORF products, using the optimized BlastN parameters: identity threshold >95%, word size = 7, e-value = 1e-5 and complexity filter disabled. ISsaga scans the input genome fasta sequence for previously annotated ISs in the ISfinder database.

For ISs not in the ISfinder database, the user must submit the newly identified ISs so that they can subsequently be semi-automatically annotated (detailed instructions can be found in the user manual in Additional file 1. For each IS identified in this step, ISsaga creates a validation report, to be further analyzed by the annotator in the validation step.

The validation step processes the result generated by the previous steps, and exports each predicted IS identified in the nucleotide step to the annotation table. This is an entirely manual procedure, where the annotator must verify each IS prediction result. This requires some IS annotation expertise, which is detailed in the user manual.

### Open source programs used in Issaga

Open source programs used in Issaga are: BioPerl, used to run the annotation, generation of the IS validation report, context map and validation [9]; BLAST (Basic Local Alignment Search Tool) [8]; EMBOSS, the EMBO Open Software Suite [22]; MySQL, a relational database management system (RDBMS) [23]; and phpMyEdit, an instant MySQL table editor and PHP code generator used to generate the annotation table [24].

## Abbreviations

DR: direct repeat; IR: inverted repeat; IS: insertion sequence; ISsaga: Insertion Sequence semi-automatic genome annotation; MIC: mobile insertion cassette; MITE: miniature inverted repeat transposable element; ORF: open reading frame.

## Authors' contributions

AMV conceived and developed ISsaga, and drafted the manuscript. PS carried out ISsaga tests and design, managed the ISfinder database and drafted the manuscript. EG carried out ISsaga tests, and annotated the eight bacterial chromosomes used in this study. VC participated in the development of ISsaga. MC participated in its design and coordination and helped to draft the manuscript. All authors read and approved the final manuscript.

## Supplementary Material

Additional file 1 **ISsaga user manual**. A detailed explanation of the use of ISsaga and instructions concerning the correct system of annotation for insertion sequences. Click here for file

Additional file 2 **Figure S1 - annotation table**. This shows a partially completed annotation table of *Acaryochloris marina *with its different fields necessary for a proper annotation. The boxes are automatically filled following validation of the ISs in the individual IS reports. Each field is clickable and editable. Click here for file
