# Supplementary material for: ISsaga is an ensemble of web-based methods for high throughput identification and semi-automatic annotation of insertion sequences in prokaryotic genomes
Source: Genome Biol. 2011 Mar 28;12(3):R30. doi: 10.1186/gb-2011-12-3-r30 (PMC3129680; doi:10.1186/gb-2011-12-3-r30)
Supplement: Additional file 1 — ISsaga user manual. A detailed explanation of the use of ISsaga and instructions concerning the correct system of annotation for insertion sequences. [file gb-2011-12-3-r30-S1.DOC]

**ISsaga Manual**

**Insertion Sequence semi-automatic genome Annotation**

**Alessandro M. Varani**

**Patrícia Siguier**

**Edith Gourbeyre**

**Jocelyne Perochon**

**Michael Chandler**

**(Version 1.0)**

**Table of Contents**

1. Introduction

1.1. Overview

- 1. What is ISsaga?
  2. Features
  3. Requirements
  4. Limitations

2. Pre-Annotation

2.1. ISsaga Welcome Page

2.2. Annotation ID number

2.3. Controlling the user account

3. Starting the Annotation

3.1. How to submit a genome file to ISsaga

3.2. Annotation and prediction & estimation page

3.2.1. Annotation of single replicons

3.2.2. Annotation of multiple replicons

3.3. Annotation Table

3.3.1. Editing the Annotation Table

3.3.2. Add a new IS

4. Semi-Automatic Annotation Steps

4.1. Semi-Automatic IS identification

4.2. Validating the Semi-Automatic Annotation (IS Validation Report)

4.3. Reconstructing a disrupted IS

5. Manual Annotation Steps

- 1. Identification and annotation of the reference IS copy
  2. How to obtain an IS name
  3. Dealing with false positives
  4. Studying the impact and role of IS using the IS ORFs Context Table
  5. Submitting newly identified IS to ISfinder

6. Finishing the annotation

6.1. Exporting the annotation to a new GenBank file

7. Annotation examples

9. Credits

**Chapter 1. Introduction**

**1.1. Overview**

The in-depth analysis of the growing number of completely sequenced prokaryotic genomes is providing important contributions to our understanding the biological world. Large-scale sequencing of prokaryotic genomes demands automation of certain annotation tasks, such as prediction of coding sequences and promoters. But automatic processes are severely limited and can lead to poor quality annotation at the DNA level. A crucial example of this is the annotation of mobile genetic elements (MGE). These have played a major role in genome evolution and contribute massively to horizontal gene transfer. A detailed and accurate analysis of MGE content and distribution would provide an important picture of the evolution of their host genomes.

Unfortunately, a majority of bacterial and archeal genome sequences deposited in the public databases are seriously compromised in their MGE annotation, particularly for one of the simplest class of autonomous MGEs found: the insertion sequences (ISs). In particular fragments of incomplete ISs which represent the scars of previous recombination events are rarely annotated but provide important information on genome evolution.

To facilitate the annotation of these elements, ISsaga provides a high-quality semi-automatic annotation system, directed to the accurate identification and annotation of ISs.

**For an extensive explanation concerning the General features and properties
of insertion sequences, see:**

1) <http://www-is.biotoul.fr/is/IS_infos/is_general.html>

2) Mahillon J. and Chandler M. (1998) Microbiology and Molecular Biology Reviews. 62 : 725-774

3) Chandler, M. and Mahillon, J. (2002) Insertion Sequences Revisited Mobile DNA II Edited by N.L., Craig et al. ASM Press 305-366

**1.2. What is ISsaga ?**

ISsaga (Insertion Sequence semi-automatic genome annotation) is a tool developed by the ISfinder Team (http://www-is.biotoul.fr/) for annotation of Insertion Sequences (IS) in prokaryote genomes. It performs automatic tasks such as transposase identification, IS family attribution and IS annotation reports. The system integrates several resources from the public domain and newly developed software capable of dealing with the different types of IS families.

This software comprises a web environment and a set of PHP/Perl scripts that manipulate data within a relational database (MySQL). The system provides forms, tables and generates reports through which users can access both biological information and load the results of their analysis onto the database.

The software includes semi-automatic and manual steps. The first, semi-automatic, module contains the protein annotation steps (transposase detection and classification) and part of the nucleotide annotation steps for pre-identified ISs. Pre-identified ISs are ISs which are already in the ISfinder database. The manual module provides the necessary tools to identify and annotate new ISs.

**1.3. Features**

The most important feature offered by ISsaga is to provide a complete IS annotation using strict rules. Other important features are:

- Provision of an IS annotation table with the rules necessary for ISs annotation (topic 3.3).
- Prediction & Estimation of the IS population in a given genome sequence.
- Identification and annotation of “partial” ISs.
- Identification and annotation of solo IRs (isolated copies of the ends, equivalent to isolated LTRs in eukaryotes).
- Full integration with ISfinder (full integration with ISbrowser is under development).
- Automatic IS-associated orf prediction from unannotated FASTA files.
- Exportation of the annotation in new GenBank or Excel files.
- Dynamically generated annotation status reports and previews.

**1.4. Requirements**

**Computer requirements:**

We recommend Firefox (2.0 or higher), Google Chrome, Safari (4.0 or higher), or Opera (10 or higher) with the latest version 6 or higher of the Java (javascript enabled) and Flash plug-in version 8 or higher. Internet Explorer may also be used but is not recommended.

**Please notify the ISfinder Team through the contact form** (<http://isteam.biotoul.fr/ISsaga/c.php>) **in the event that you encounter problems with program compatibilities, capabilities and bugs.**

**System Requirements:**

***User registration:*** To ensure security and organizational requirements, the user must sign an online agreement concerning the conditions of ISsaga use (<http://issaga.biotoul.fr/ISsaga/agreement.php>). This is accessible on the user registration form. The user must create an account, register and choose a user-name and password using the form shown in figure 1.1. The user must complete all fields marked with an asterisk. There is also an optional field for including the list of genomes which are to be annotated. Although optional, this will avoid multiple annotations of the same genome by different laboratories. The account approval time is up to 72 hrs.

**
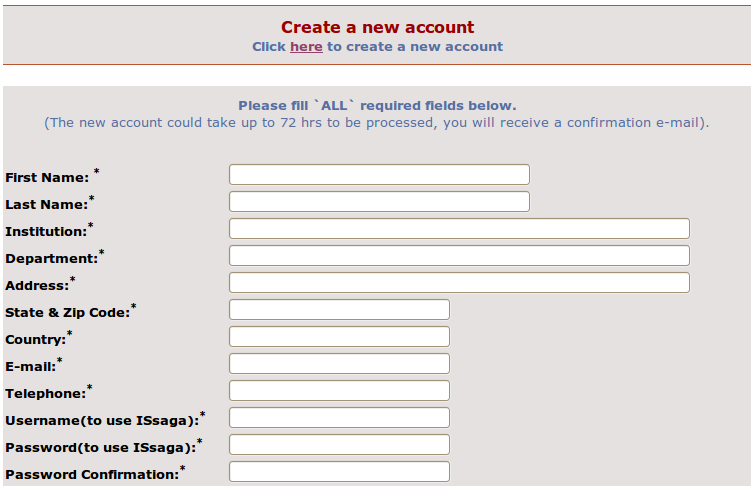
**

**Figure 1.1.** Creating a new account. Detail of part of the account form.

***Input genome files:***

ISsaga accepts pre-annotated **GenBank** files (.gbk) and **FASTA** nucleotide files (.fasta). It will also accept FASTA protein files (.faa) but only together with the corresponding FASTA nucleotide file.

The system will perform automatic IS-associated orf identification using an optimized transposase gene model (provided by ISfinder) only for ".fasta" input files alone. If provided with the corresponding ".faa" it will consider the ".faa" file as the pre-annotation.

The **recommended** genome input file for ISsaga is the GenBank file, because this file format normally includes pseudogene annotations.

**The annotator must keep in mind that the quality of the previous annotation (for pre-annotated submissions) is important for good automatic results. For this reason, we have introduced a user validation step for each piece of information extracted.**

Figure 1.2 shows an example of a GenBank file. Information about GenBank file standards can be found directly at: <http://issaga.biotoul.fr/ISsaga/f_format.php> or through the "Start Annotation" button under the "File Format" link. Bacterial and Archaeal GenBank files can be downloaded from: ftp://ftp.ncbi.nlm.nih.gov/genomes/Bacteria

**
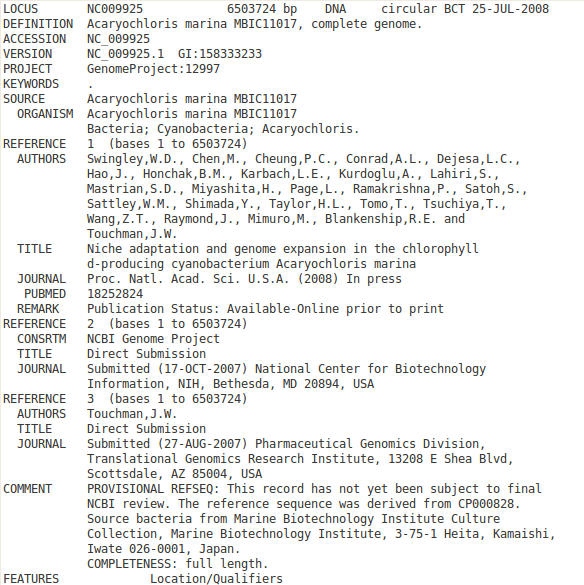
**

**Figure 1.2.** Example of GenBank file (in detail the header of the GenBank file)

**1.5. Limitations**

ISsaga has been developed for high-quality IS annotation and does not provide annotation tools for other mobile genetic elements such as prophages and integrons. Although the system provides some automatic steps, it does not provide a complete automatic IS annotation. Every automatic result must be inspected and curated by the user.

The accuracy of the annotation depends critically on the quality of the pre-annotation (for GenBank files). We have experienced several problems related to prior transposase annotation such as the absence of correct start and stop codons or of the correct transposase (e.g. where full length transposases are generated by translational frameshifting as in several IS families, Topic 5).

The IS prediction and estimation is not 100% accurate. Internal validation tests guarantee accuracy between 60% to 95%, depending of the submitted genome and the coverage of ISfinder database.

Other limitations are that, for FASTA input, the system does not provide transposase pseudogene identification and, at present, ISs with more than 3 ORFs will not be accepted in the semi-automatic steps. The user must manage these cases manually.

**Chapter 2. Pre-Annotation**

Having obtained a username and password the user is then able to access the system. The first screen is the **ISsaga Welcome Page.**

**2.1. ISsaga Welcome Page**

The upper menu (figure 2.1) provides four options: "HOME" (a link back to the ISsaga Welcome page); “YOUR ACCOUNT” (a link to the user account administration - Topic 2.4); “ABOUT” (a link to the general information and the ISsaga manual); and “CONTACT” (a link to the contact and feedback form).

**
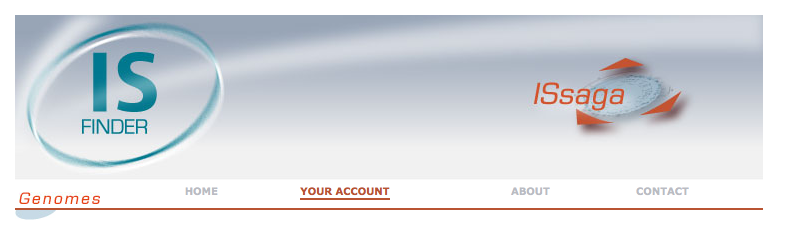
**

**Figure 2.1** The Upper Menu

The middle section of the page (figure 2.2) includes three buttons: **Start the annotation** using a genome file provided by the user; **File Conversion Tool** providing options to convert different genome input files to a format accepted by ISsaga (Topic 3).

**
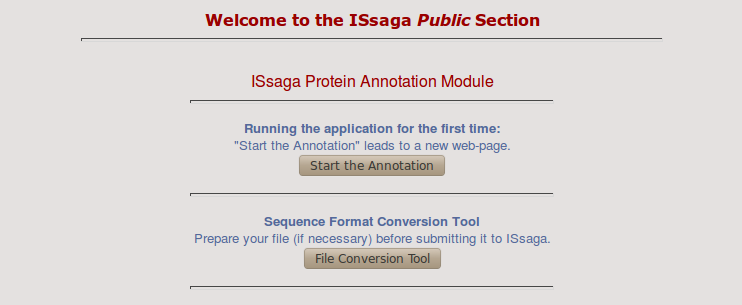
**

**Figure 2.2.** Middle Menu, Getting started

Each annotation project has its own ID number. If the user has previously stored data in the ISsaga genome server, this can be accessed directly using the previously stored ID number (figure 2.3).

**
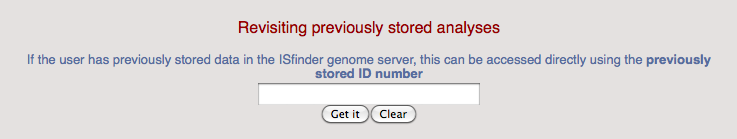
**

**Figure 2.3.** Revisiting previously stored analyses.

**2.2. Finding your annotation ID number**

The ID number is composed of 19 digits (e.g. **1608101108830244062).** This is displayed at the bottom of each submitted Project (figure 2.4) or can be accessed in “YOUR ACCOUNT” link. (Topic 2.3).

**
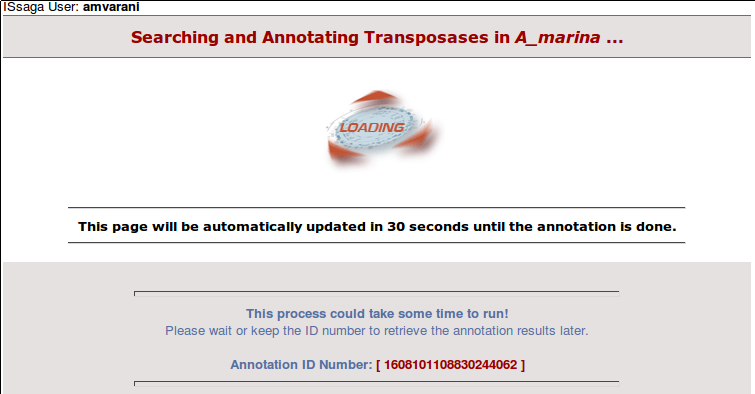
**

**Figure 2.4.** Locating the annotation ID number

**2.3. Controlling the user account**

The link “YOUR ACCOUNT” (figure 2.5) provides the user with the following options:

“Verify Annotation Status” opens a new window containing the information for each project submitted by the user (figure 2.5b). It shows the project name, ID number, whether it has been finished, the date when it was finished and whether it has been validated (Topic 6). Clicking on the ID number will open the annotation.

"Changing account details" provides an option to change the password, address and contact information.


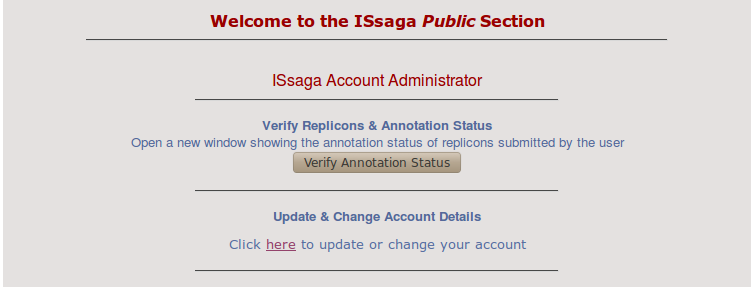
**a)**

**b)**


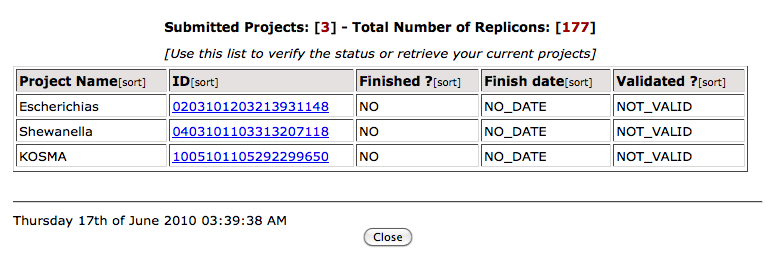


**Figure 2.5.** **a**, Account and project management window. **b**, example of the pop-up window with the current submitted projects by the user/annotator.

**Chapter 3. Starting the Annotation**

**3.1. How to submit a genome file to ISsaga (protein annotation step)**

To start the annotation using a pre-annotated genome file (GenBank) or a genome FASTA file, the user must complete the required fields in the “Start the Annotation” page (figure 3.1) which is accessed from in the ISsaga Welcome page (figure 2.1).

The user must: 1) provide a **project name** (up to 16 characters); 2) specify the **genome input file** format (Topic 1.4); 3) choose **how many replicons to annotate** (the default system accepts up to 10 replicons for more experienced users); 4) specify **the organism name**, **NC number** (respecting GenBank format) and **upload** the genome input file (GenBank or FASTA files) for each replicon specified; 5) **Send/Run** starts the semi-automatic annotation by initiating the protein annotation steps (transposase detection and classification) and the first level of nucleotide annotation steps.


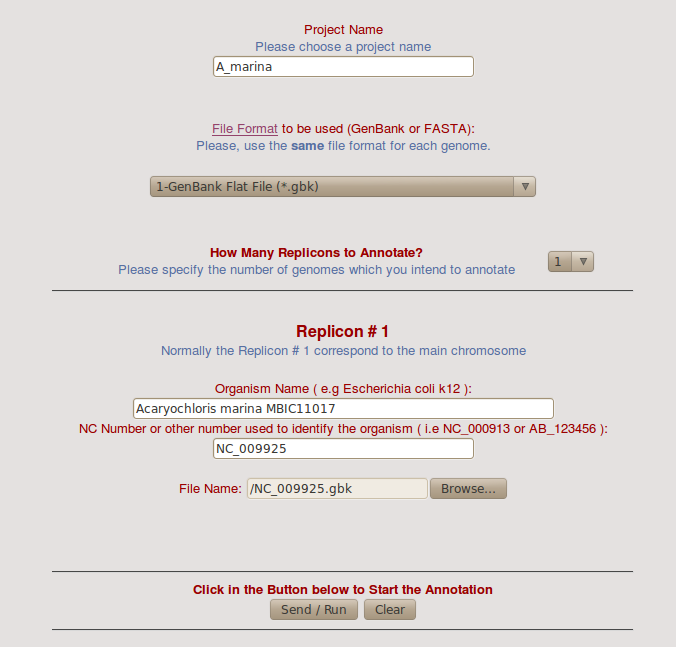


**Figure 3.1.** Starting the annotation. Example showing all required fields to start the annotation

The annotation time depends on three factors: the length of each submitted replicon; the number of putative IS-associate proteins identified; and the average load on the ISfinder server. For example this first step took 16 minutes for the ***Acaryochloris marina* MBIC11017** genome (~6.5 Mb and 277 IS-associated orf) on our servers.

The “loading page” is displayed during loading and running (figure 3.2). This shows the annotation ID number at the bottom. The user can close this window while performing other tasks, and retrieve the results later using the “YOUR ACCOUNT” link (Topic 2.4), or wait for the annotation results which will be shown automatically at the end of this process (figure 3.3a). **Note that the user can only run one project at a time.**

During the analysis the input genome is compared with the ISfinder database. In the case of a Genbank file all pre-annotated proteins (including pseudogenes) are extracted and compared using BLASTp and BLASTx against the ISfinder protein database. For FASTA nucleotide ".fasta" input files the system will perform orf predictions using the GLIMMER software optimized with an IS associated orf training-set obtained from ISfinder (note that in this case pseudogenes will not be detected).

When this initial step is finished, an annotation page will appear automatically.

**
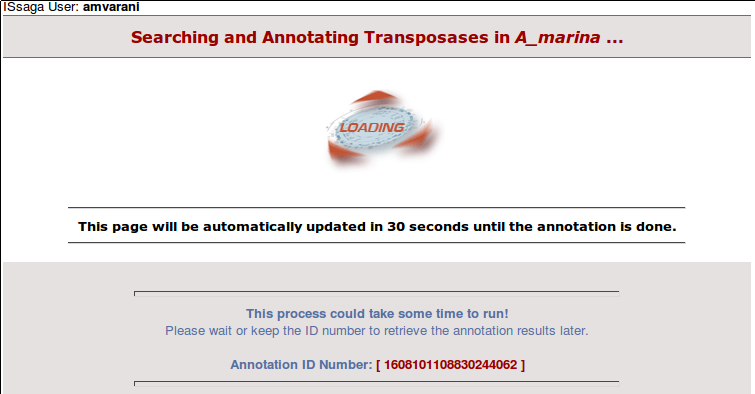
**

**Figure 3.2.** Loading a genome in ISsaga: the loading webpage with the annotation ID number.

**3.2. Annotation and Prediction & Estimation page**

The annotation page (figure 3.3a) contains a colour coded pie chart showing the distribution of all predicted IS (indicating the number of regions potentially carrying an IS of a given family as a percentage of all potential IS-carrying regions in the entire replicon). Below the pie chart is a summary of the number of potential IS-associated orfs, the estimated number of complete and partial orfs, pseudogenes and orfs of unknown status as well as the predicted total number of ISs and number of different ISs. These will include both transposases and accessory genes together with possible false positives (orfs possessing the minimal threshold of similarity to true transposases, Topic 5.3).

The pie chart provides an estimation of the possible IS content of the genome (expressed as a percentage of the total number of potential ISs). Where an IS is a member of a known family whose members carry more than one orf, the programme considers this as a region which could contain a single IS (e.g. the two proteins IstA and IstB encoded by IS*21* would be considered as a single IS occurrence). However, there is no distinction between complete and partial elements. These will be corrected in the following steps (Topic 5). Each pie chart segment is clickable and leads the user to a section of ISfinder containing general information about the IS family in question.


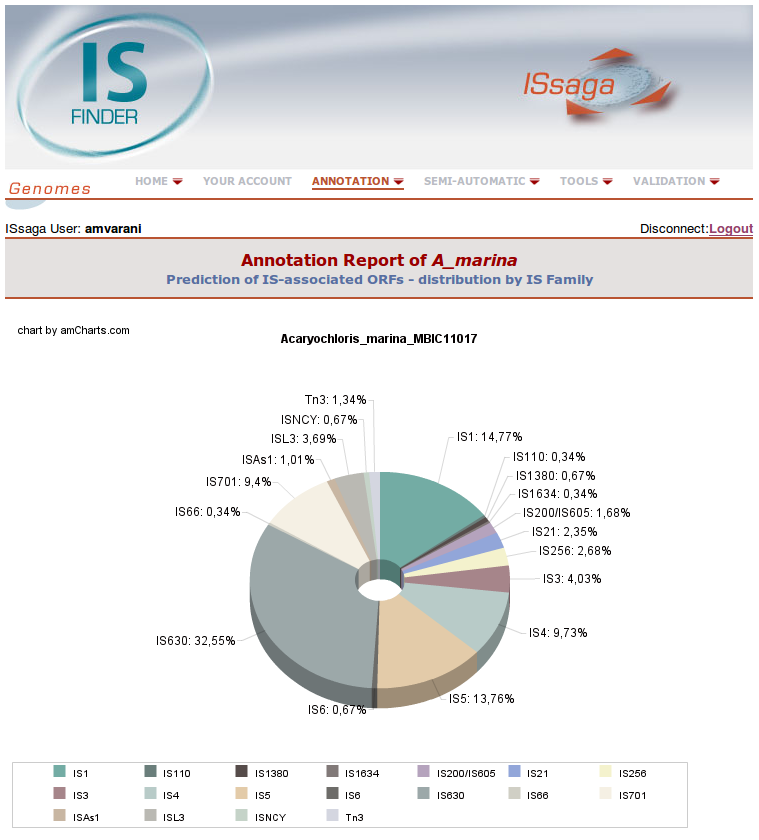

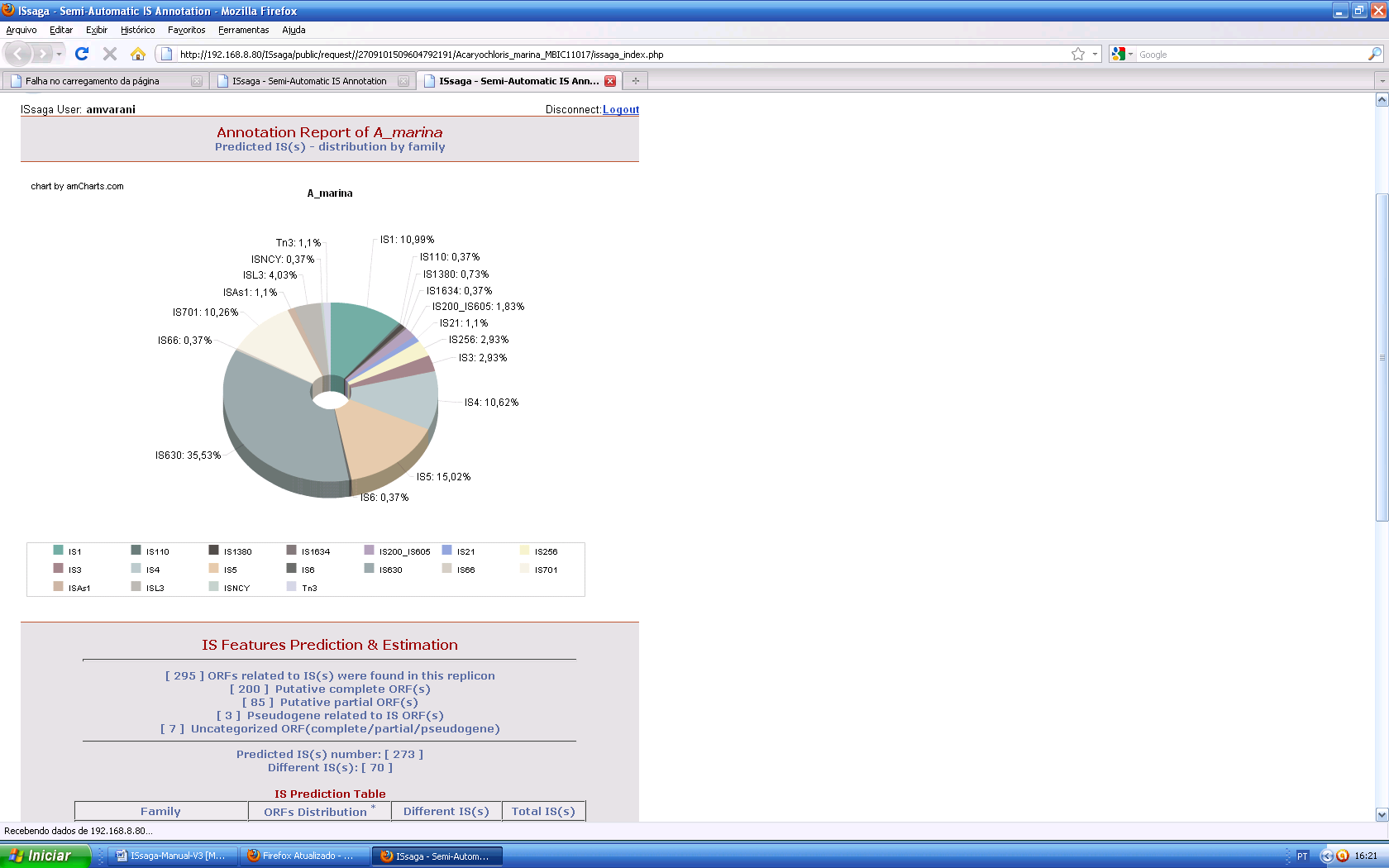


**Figure 3.3a.** Annotation page for a single replicon showing the four main options, the pie chart and the first part of the IS population estimation & prediction.

The results below the pie chart (figure 3.3a) present an estimate of the total number of IS-associated orfs, and a prediction of the number of complete and partial orfs, pseudogenes and uncategorized / unknown orfs. It also provides an estimate of the total number of ISs and of the number of different predicted IS (the fasta nucleotide sequence of each predicted different IS can be accessed through the SEMI-AUTOMATIC drop-down menu). For single or individual replicons the system also shows a table (figure 3.3b) containing the number of complete and partial orfs, pseudogenes and unknown orfs, distributed by each identified family, associated with the predicted number of total and different ISs.


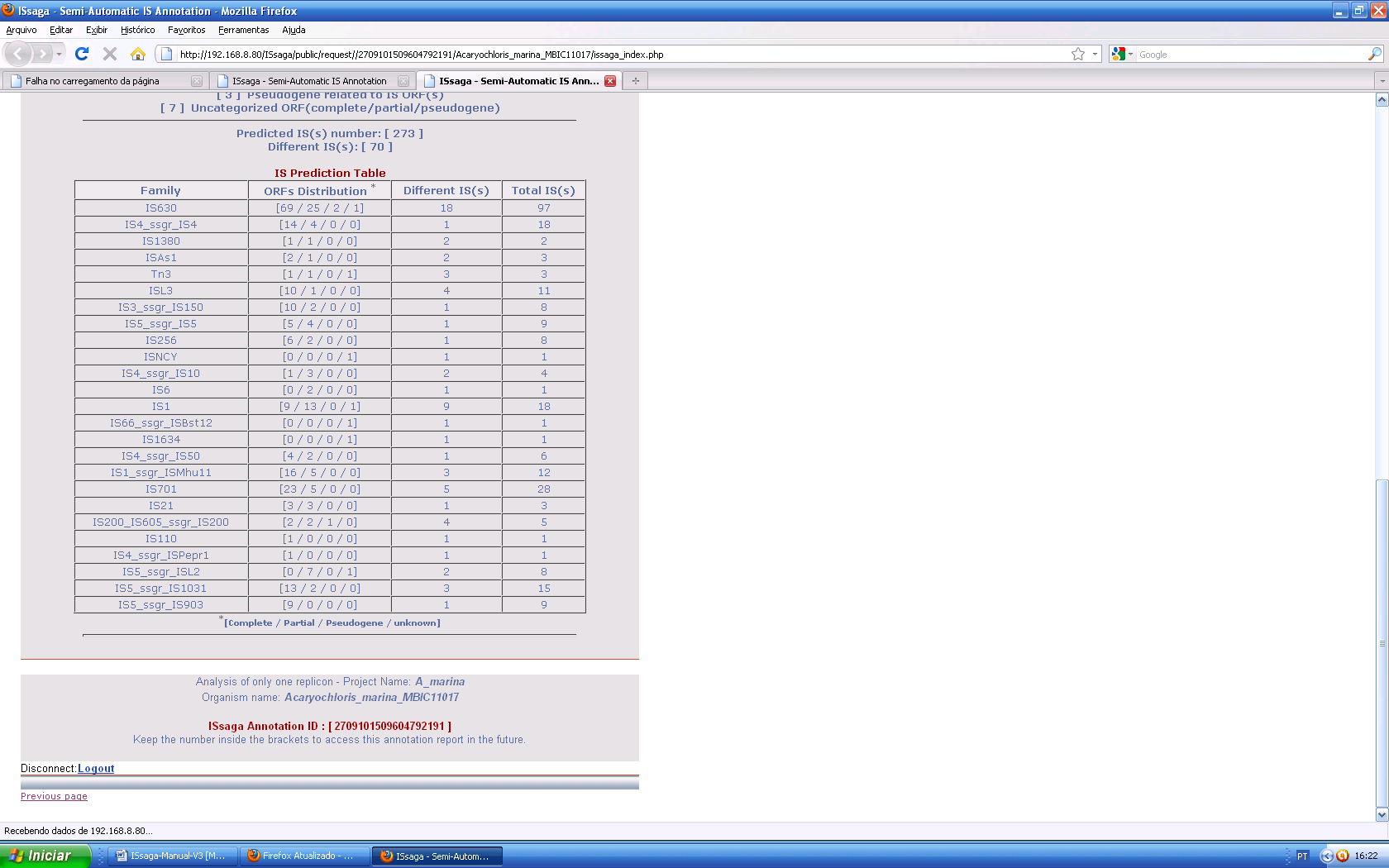


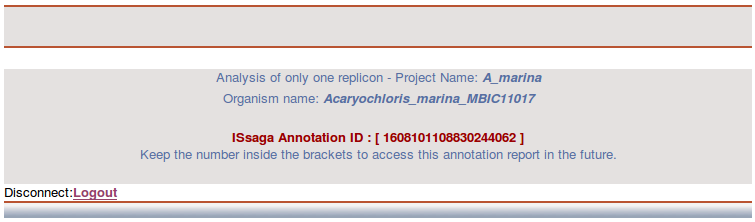


**Figure 3.3b.** Annotation page for a single replicon showing the prediction table with the IS family distribution.

The annotation page menu also includes four main options with drop-down menus: ANNOTATION; SEMI-AUTOMATIC; TOOLS; and VALIDATION (figure 3.3a). To continue the annotation process, the user must access the ISsaga annotation system via the "annotation menu" which contains a set of tools to facilitate automatic and manual annotation.

The annotation page for projects with single or multiple replicons differ slightly.

**3.2.1. Annotation of single replicons**

**ANNOTATION:** Provides the user with the option of inputting information and retrieving annotation results via the drop-down menu. This comprises:

- ***Annotation Prediction:*** Allows the user to access the original pie-chart showing transposase distribution.

***- Annotation Table:*** This table will contain the protein information generated by the loading process together with genome coordinates. It allows the user to manually input additional annotation features and to validate the results for each identified IS. This section is one of the most important features and will be documented separately (Topic 3.3).

***- Extract Annotation:*** Exports the annotation to generate a file in Excel or Genbank format (Topic 6.1)

***- ISbrowser Preview:*** Dynamically generates an IS annotation summary showing the distribution as a circular genome map using the CGView tool.

***- Annotation Status:*** Dynamically generates charts showing annotation progress (Number of annotated ISs, IS distribution by type and family).

***- IS ORF Context:*** Contains a table showing the genome context of each IS-associated orf identified. It allows the user quick access to the 1000bp nucleotide sequence upstream and downstream of each predicted IS-associated orf, and to input additional information. It should be used together with the ***Annotation Table*** to verify the role of the IS in gene disruption (Topic 5.4).

**SEMI-AUTOMATIC:** Provides links related to semi-automatic IS annotation / identification.

***- List Annotated ISs:*** Displays the predicted ISs in the given project, using previously identified ISs deposited in the ISfinder database. All pre-identified ISs are associated with a link to the IS report, a table including all features of a given IS identified on the replicon (Topic 4.2).

***- Predictor & Flanking Region Extractor:*** Opens a tool which is used to determine the number of copies of a given IS and their flanking regions (insertion sites) (Topic 5.1).

***- Submit Reference IS copy:*** Opens a tool to submit a newly discovered IS (not present in ISfinder) as a reference copy which can then be used to annotate additional copies in the genome (Topic 4.2).

***- Extract IS(s):*** Opens a tool to extract different features (nucleotides, orfs, IRs, DRs) of all genomic copies of the annotated IS in FASTA format.

***- Construct Ref. IS Copy:*** Opens a tool which is used to facilitate creation of a reference IS copy for ISs which are not already present in the ISfinder database (Topic 5.1). It comprises four tables with information about the candidate IS-associated orfs, and the putative regions which could carry an IS identified in the project: *Pre-identified Different ISs* shows the nucleotide fasta sequence of each different region which could carry an IS. Each fasta sequence contains the main core of a putative IS element together with flanking sequences which may include both IS ends and the DRs. However, these must be annotated manually using the **TOOLS** menu. This is helpful in determining the **reference IS copy** for submission (Topic 5). *The Transposase Hit Table* shows the best hits (>95% identity) between the identified orf and other copies in the genome. It is used to identify orfs of other copies of the same IS; *Transposase Family* gives the IS family of each candidate IS-associated orf identified and the first Blast hit in ISfinder. It can be used to examine the variability of orf sequences between the various copies of a given IS and differences between different members of the same IS family; *Singlets* shows candidate IS-associated orfs with no matches against other IS-associated orfs in the same project. This identifies unique single copy ISs but may also include false positives.

**TOOLS:** This includes a set of web tools to be used to manually identify the reference IS copy (Topic 5.1).

***- IS Notepad:*** Stores information (nucleotide sequence, IR, DR etc) necessary for identification and definition of the reference IS copy. It provides a template for defining all features of the reference IS in a format which can be submitted to ISsaga (and ISfinder). This can be filled-in manually.

***- Replicon BLAST:*** BLAST of a putative IS-carrying sequence (nucleotide or protein) against the project genome sequence. This permits identification of correct ends, partial copies and the copy number of the IS with the genome.

***- BLAST 2 Sequences:*** Allows positioning of nucleotide coordinates of an orf on those of a complete IS

***- ISfinder BLAST:*** Compares the test sequence against the IS Finder Database

***- ORF Finder:*** Searches for orfs in a given sequence. Can be used to identifiy unannotated IS-associated orfs.

***- Filter DNA:*** Removes non-DNA characters from text. Use this program when you wish to remove digits and blank spaces from a sequence to make it suitable for other applications.

***- Revert DNA:*** Converts a DNA sequence into its reverse, complement, or reverse-complement counterpart. This is used to determine the IS orientation.

***- Translator:*** Translates DNA sequences in the six frames. This can be used to identify potential programmed translational frameshifts which occur in some IS families (e.g. IS3)

***- View Ends:*** Verifies and identifies the Inverted Repeats in the reference IS copy.

***- Sequence Extractor:*** Extracts a user-defined DNA region. This can be used to extract a region of DNA which carries a potential IS copy which can then be used in the "***Replicon BLAST***".

**VALIDATION:** When finishing the annotation, the user has the option of sending the results to the ISfinder curators for quality control and validation. This option is mandatory prior to its submission to ISfinder and subsequent inclusion in ISbrowser.

***- Submit a new IS to ISfinder:*** If the user identifies new ISs during the annotation procedure (as expected for most new genomes), as agreed in the conditions for ISsaga use, these **must** be submitted to ISfinder using the submission form.

***- Finish the Annotation:*** Submits the annotation for Validation. The user should download a copy as an Excel or GenBank file using the "extract annotation" in the ANNOTATION drop-down menu.

**3.2.2. Annotation of multiple replicons**

The **Annotation page** for projects with multiple replicons includes a pie chart containing the sum of predicted IS for all the replicons. A list of "Replicons analysed" appears below with a direct link to the content of the individual replicons (also in the form of a pie chart). The other important differences are located in the drop-down menus.

**ANNOTATION menu:** This provides both a global report containing all replicons being analysed and individual reports for each replicon. The user can choose between these options.

***- Replicons Analysed:*** Provides the list of replicons, each with a link to its individual pie chart and individual replicon annotation page. The ANNOTATION and SEMI-AUTOMATIC drop-down menus for these individual replicons are as described in **3.2.1.**

- ***Global Annotation Prediction:*** This accesses the original global pie chart containing the sum of orfs for all replicons.

***- Annotation Table:*** This allows the user to access the annotation tables of each individual replicon independently.

***- Annotation Status:*** This permits visualisation of the progress of global annotation (similar to that for individual replicons, **3.2.1**).

**Note:** Single replicons viewed from the multiple replicon menu have slight differences from the single replicons menu. These are found in the *Global Annotation Prediction, Annotation Prediction, Annotation Table, Extract Annotation, ISbrowser Preview, Annotation Status and IS ORF Context* sections of the **ANNOTATION menu.**

**3.3. Annotation Table**

The annotation table (figure 3.4) is composed of lines each representing a candidate IS-associated orf and columns including different types of information necessary to describe and annotate the IS. The lines are placed in order of the orf position along the genome. The annotation table is dynamically generated and fully clickable. The initial genome loading procedure will generate an annotation of candidate IS-associated orfs which will be automatically included in this table. **Each line of the table must be verified by the user** to ensure the quality of the annotation.

**
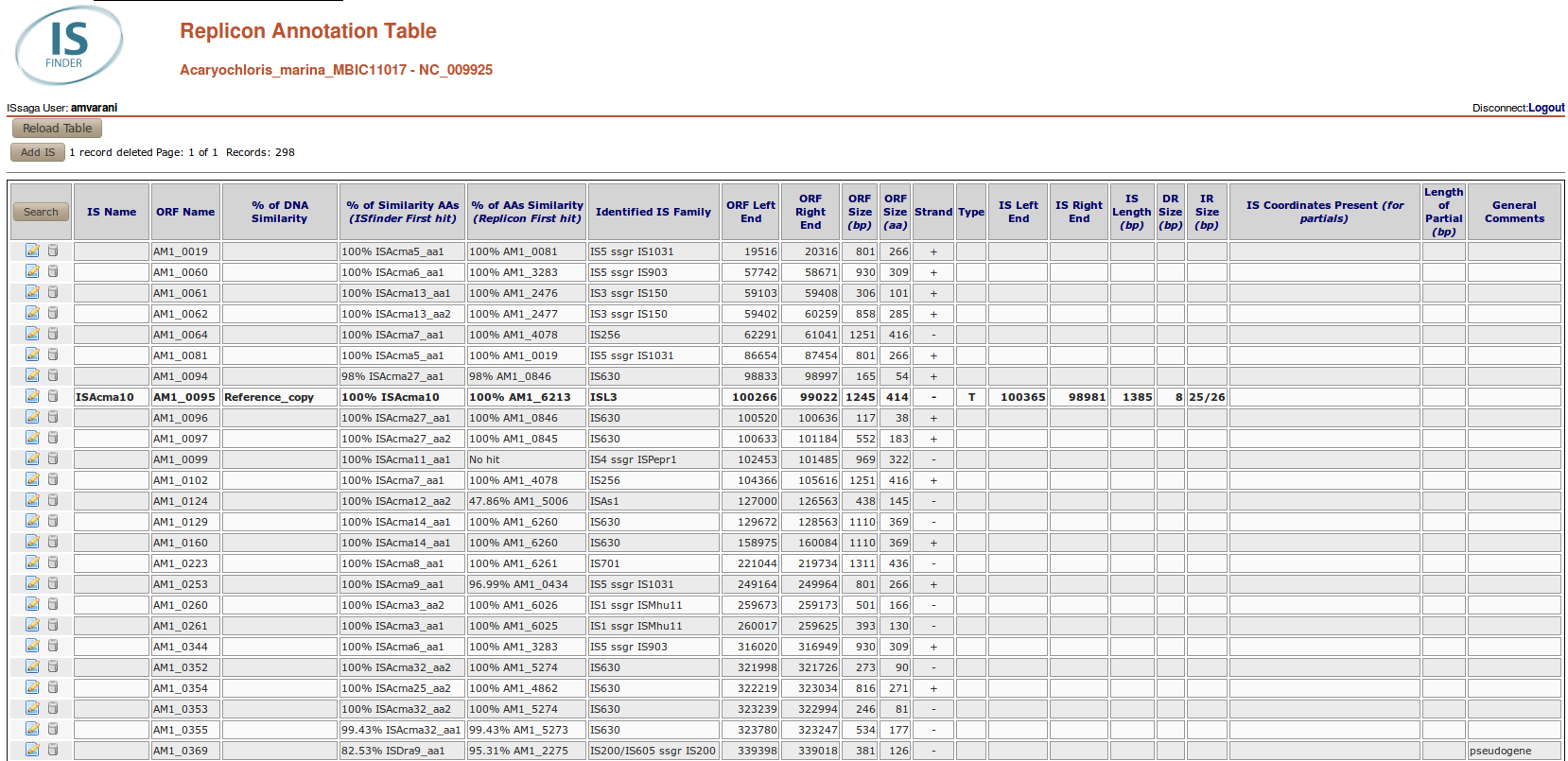
**

[Part of this picture has been deleted for brevity]


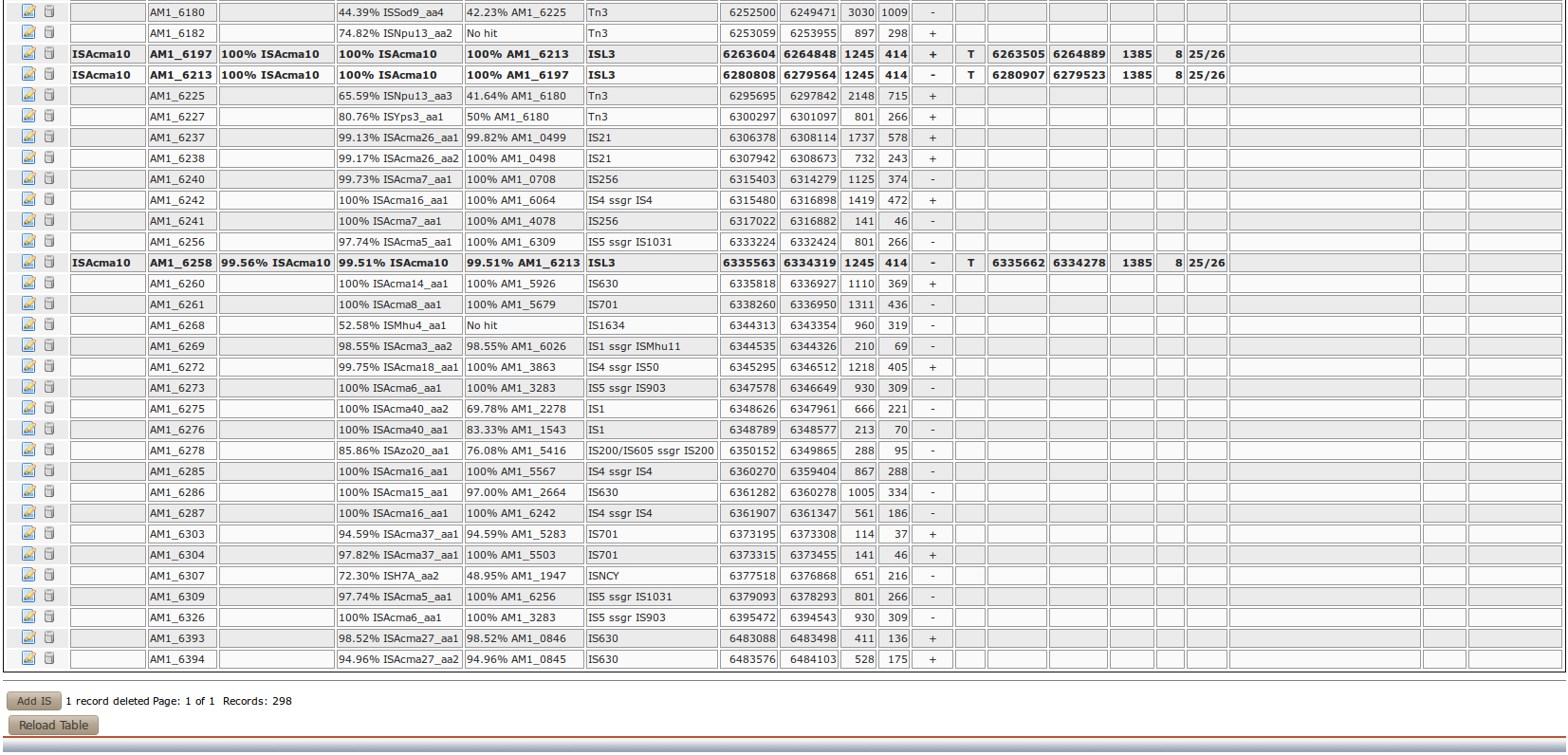


**Figure 3.4.** Annotation table displaying the information related to the annotation.

The annotation table is composed of the fields:

- IS Name: Name of the reference IS. This **must respect the ISfinder nomenclature standards (Topic 5.1).**

- ORF Name: Predicted ORF name automatically generated during protein annotation steps and extracted from the previously annotated GenBank or FASTA protein file or, for FASTA nucleotide files, generated by the GLIMMER procedure (Topic 3.1.).

- % of DNA Similarity: DNA similarity with the reference IS sequence.

- % of Similarity AAs (*ISfinder First Hit*): Protein Similarity with the first hit in the ISfinder Database

- % of AA Similarity (*Replicon First Hit*): Protein Similarity with the first hit in this replicon.

- Identified IS Family: IS family detected by the automatic annotation.

- ORF Left End: Left (5'; N-terminal) position of the orf in the replicon.

- ORF Right End: Right position in the replicon.

- ORF Size (*bp*): Size in base pairs (including the stop codon).

- ORF Size (*aa*): Size in aminoacids.

- Strand: ORF Orientation.

- Type: This describes the functions of the IS-associated orf. [ T ] transposase, [ P ] passenger genes, [ A ] accessory gene or [ ND ] not determined.

- IS Left End: IS left coordinate in the replicon. (Generally the left end is defined as that upstream of the transposase orf).

- IS Right End: IS right coordinate in the replicon.

- IS Length (bp): IS length in base pairs (including Inverted Repeats, but excluding the Direct Repeats).

- DR Size (*bp*): Direct repeat length in base pairs. [Not all ISs have flanking DRs]

- IR Size (*bp*): Inverted repeat length in base pairs. [example: 22/30, 22 matches in 30 nucleotides] [Some particular ISs of some families do not have flanking IRs]

- IS Coordinates Present: This applies only to **Partial ISs** - Coordinates of the partial IS. [example: 100366 (218) - 101333 (1185)]

- Length of Partial (*bp*): Partial IS size in base pairs.

- General Comments: Space for including observations regarding annotation. The automatic annotation process may generate automatic comments between brackets. Comments which are not in brackets have been included automatically from the original GenBank file.

Clicking on completed fields in the column "ORF Name" will open a non-editable page describing the orf (figure 3.5). This shows the type of IS-associated orf (transposase, passenger genes, accessory gene or not determined) and the previous annotation together with the flanking orf content and the amino acid sequence (If the gene is a pseudogene the nucleotide sequence will appear).


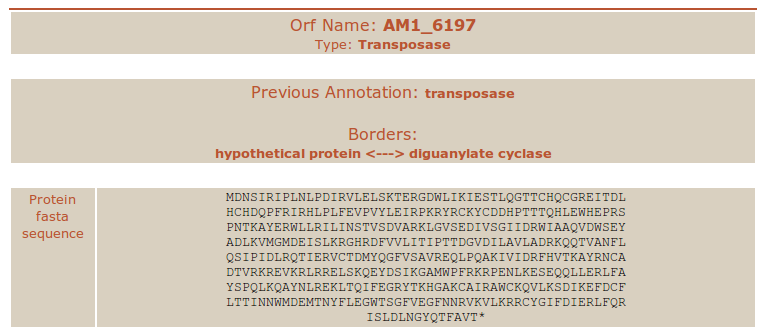


**Figure 3.5**. Link to IS overview page "Orf Name" in the annotation table

Clicking on any other field containing information will open an editing page with more detailed information for the given line (figure 3.6). This can be used to edit information and can also be accessed from the left most column of the table. The user must input additional information concerning the IS nucleotide sequence (complete, partial and solo IRs) for each IS identified. This can be performed by hand or with the tools provided in the SEMI-AUTOMATIC drop-down menu on the Annotation page. The left hand column allows the user to change information on each line or delete the line.

**3.3.1. Editing the Annotation Table**

The editing page (figure 3.6) includes all headings listed above together with additional headings:

- *Freeze Line:* blocks any information in this line introduced by hand and prevents overwriting by subsequent automatic steps performed in generating the ***IS Validation Report*** (Y for yes, N for no).

- *Previous Annotation:* Previous ORF annotation for comparison purposes.

*- IS Structure:* [ C ] complete, [ P ] partials, [ SIR ] solo IRs [ ND ] Not determined.

*- Disrupted and/or Broken IS ?:* Occurrence of **disrupted ISs** (Y for yes, N for no and ND not determined [solo IRs].

- *Disrupted IS ID:* Each reconstructed IS has an unique ID (Topic 4.3).

- *Disrupted IS Slice:* Tags each slice of the disrupted IS (Topic 4.3).

*- Number of ORFs:* Number of Open Reading frames in the detected IS(s).

*- False Positive Prediction:* Set YES for false positive IS-associated orf prediction.

*- IS ORF(s) List:* ID of each ORF within the IS (e.g. AM1_0019). Partial ISs with no ORFs will appear as `not found`.

*- ORF protein sequence:* protein sequence of the orf in Fasta format.

*- IS fasta sequence:* IS Fasta DNA sequence (with IRs but without DRs)

*- IS Left End:* Left end of the element. [For IR identification - Some ISs do not possess IRs ]

*-IS Right End:* Right *inverted* end of the element [For IR identification - Some ISs do not possess IRs ].

*-Insertion Site:* Insertion site with the presence or absence of DRs [Only shown in ***complete ISs***].


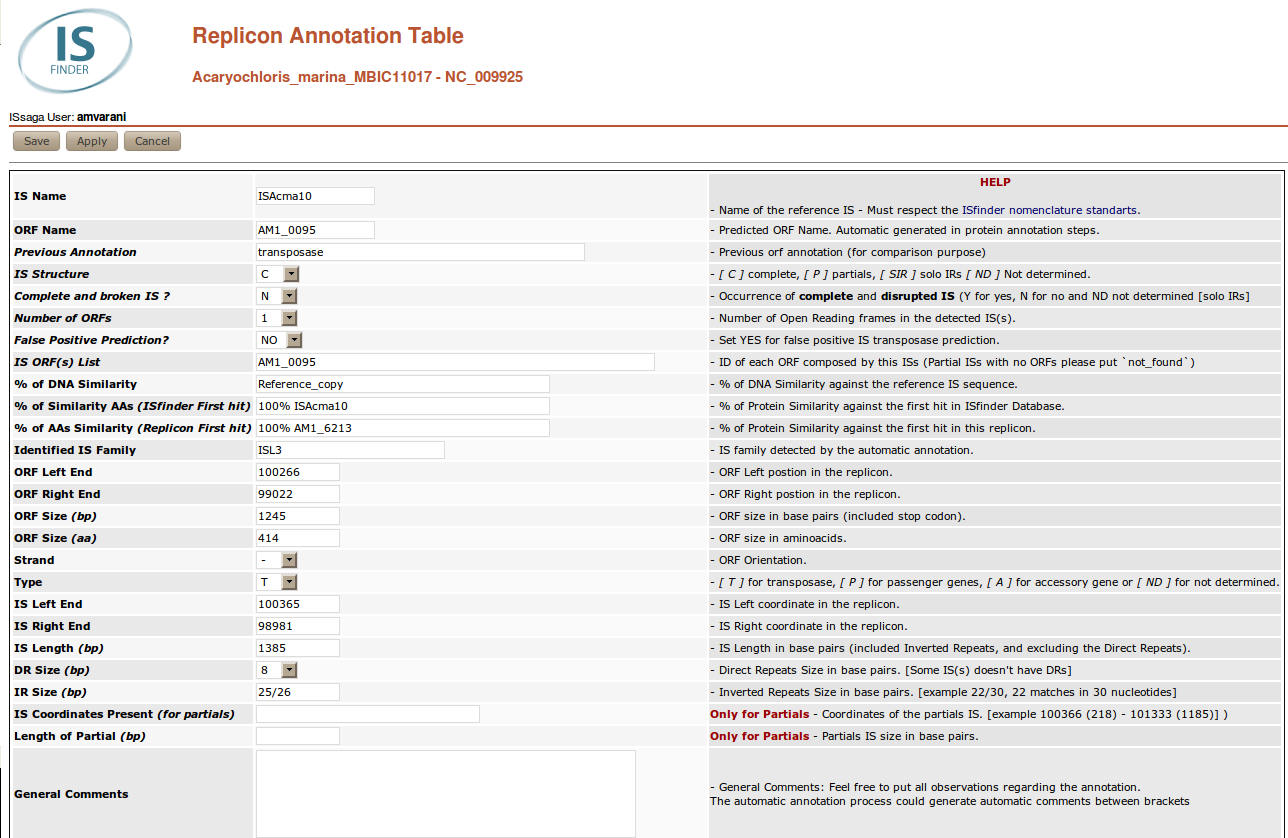


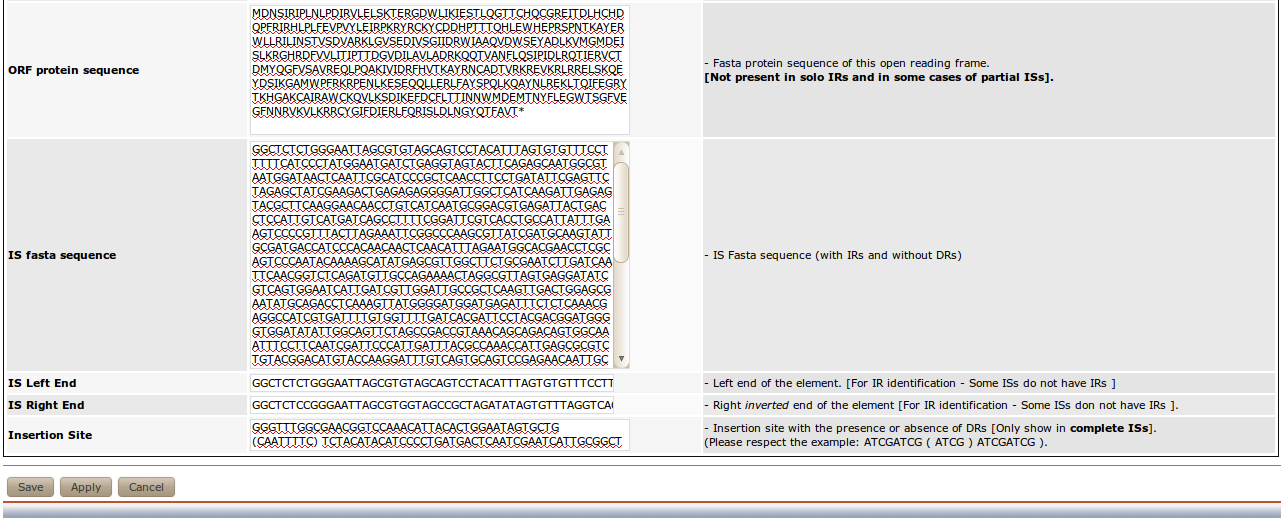


**Figure 3.6.** Annotation Table with editable fields.

**3.3.2. Add a new IS**

Clicking on the button Add IS in the **Annotation Table** (upper figure 3.4) will open an editing page which includes all headings listed above together with an additional heading:

- *Feature Colour:* Choice of a colour for a given IS which is optional but is useful to distinguish all copies of one IS from other ISs in the annotation table (default is black).

**Chapter 4. Semi-Automatic Annotation Steps**

**4.1. Semi-Automatic IS identification (nucleotide annotation step)**

ISsaga provides a semi-automatic IS identification using ISs deposited in ISfinder. The system performs an IS pre-identification during the protein annotation steps, and permits initiation of the annotation of all pre-identified ISs. The user can access this information in "***List Annotated ISs***" in **SEMI-AUTOMATIC** from the drop-down menu. Figure 4.1 shows an example of a project with pre-identified IS.

**
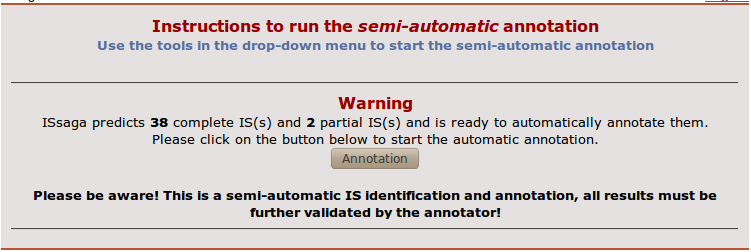
**

**Figure 4.1.** Example of project with pre-identified ISs.

If the user clicks on the “Annotation” button, the system performs a semi-automatic annotation of each identified IS. At the end of this an “annotation validation report” is generated for each IS (Topic 4.2). During this process the system shows a loading webpage (figure 4.2) and the user can close the browser to retrieve the results at a later time.

**
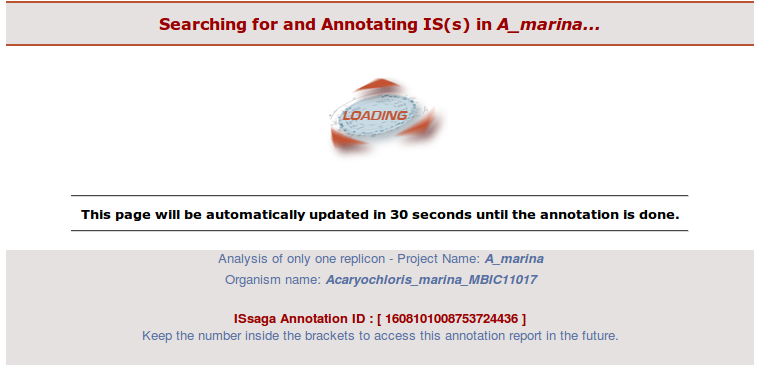
**

**Figure 4.2.** Searching and annotating the pre-identified ISs. Example of the loading page.

The user must wait for the end of the annotation when the system will show a new page indicating the replicon and containing a list of predicted ISs (figure 4.3) each with a link to its IS validation report (**4.2.**), and the IS family with a link to the example in the ISfinder database (figure 4.3).


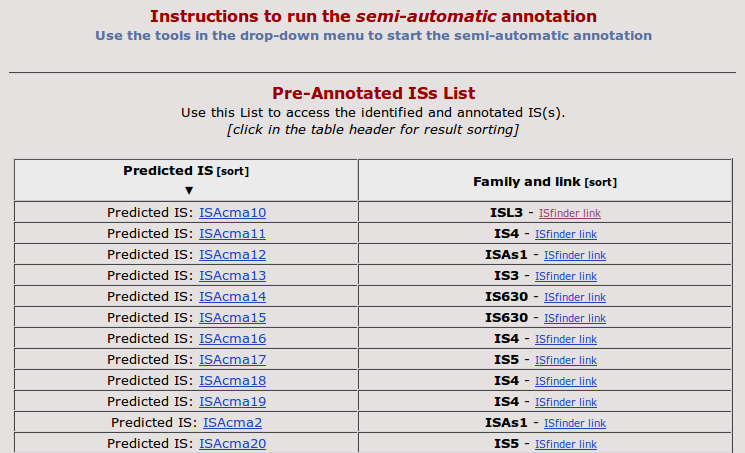


[Part of this picture has been deleted for brevity]


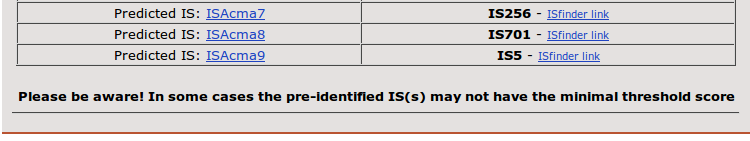


**Figure 4.3.** Table with the list of the previous identified ISs in a Single Replicon project. The first column shows the link for each IS report, and the second column show the link for the reference element in ISfinder.

**4.2. Validating the Semi-Automatic Annotation (IS Validation Report).**

The IS Validation Report (figure 4.4) is the interface which exports the annotation information to the Annotation Table. It is accessed from the IS list obtained from the pre-annotated list (figure 4.3). The report shows the prediction of complete and partial ISs and of solo IRs. There is also a graphic display showing the relative position of each IS on the chromosome with links for each BLAST result. Each line in this report includes a checkbox for user validation. Once validated, the information is automatically loaded into the annotation table.

The report is divided into **three sections:**

-**Section 1 (figure 4.4a)**: Graphical alignment which indicates the relative position of each IS with BLAST links for IS nucleotide and proteins and links to alignments of IS nucleotide and proteins. It also permits choice of a colour for a given IS which is optional but is useful to distinguish all copies of one IS from other ISs in the annotation table (default is black). This is particularly important when dealing with genomes with high IS content.

-**Section 2 (figure 4.4b)**: Nucleotide prediction which provides nucleotide coordinates of complete and partial ISs and solo IRs identified. The user must provide the inverted repeat (IR) size for each complete IS, and, using a pull-down menu, choose the IS copy which will be used as the reference. The system automatically detected slices of partial ISs which could be reconstructed as an entire IS (more details in Topic 4.3). This section also provides a list of insertion sites which can be used to define the flanking direct repeats (DR).

-**Section 3 (figure 4.4c)**: Protein prediction shows each identified orf. The user must provide the function of each protein (transposase, passenger or accessory gene) using a pull-down menu. To correct errors in a previous annotation, the option “replace coordinates” replaces the protein coordinates in the annotation table with those provided in the IS Validation Report.

**
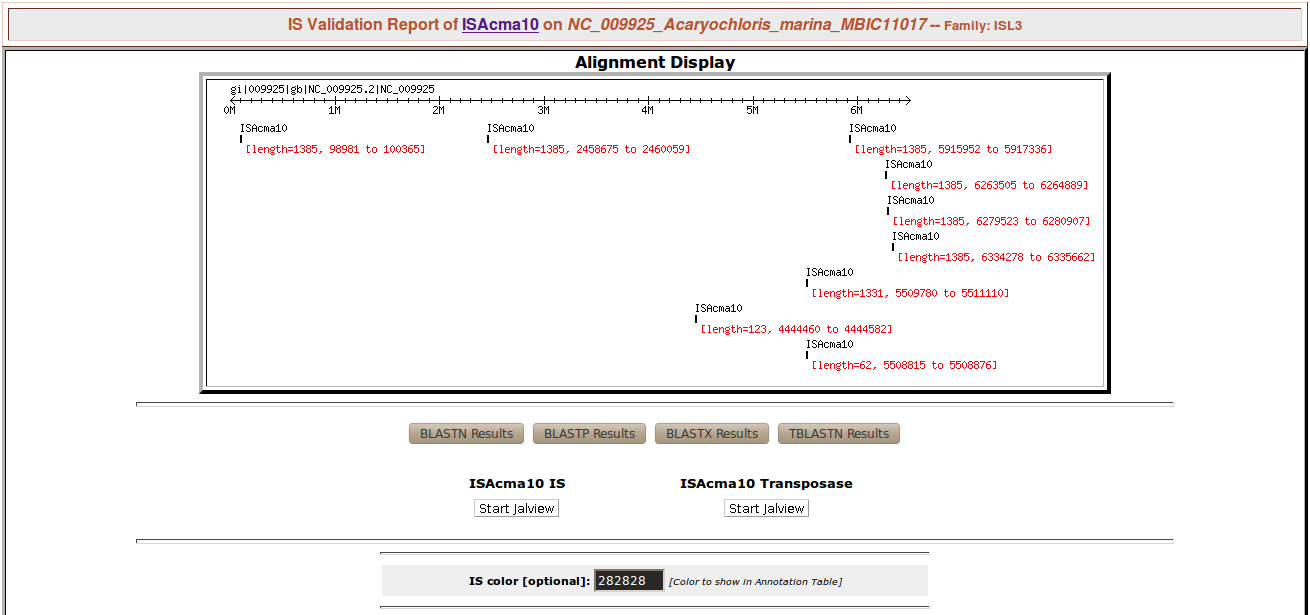
**

**Figure 4.4a**: Graphical alignment

**
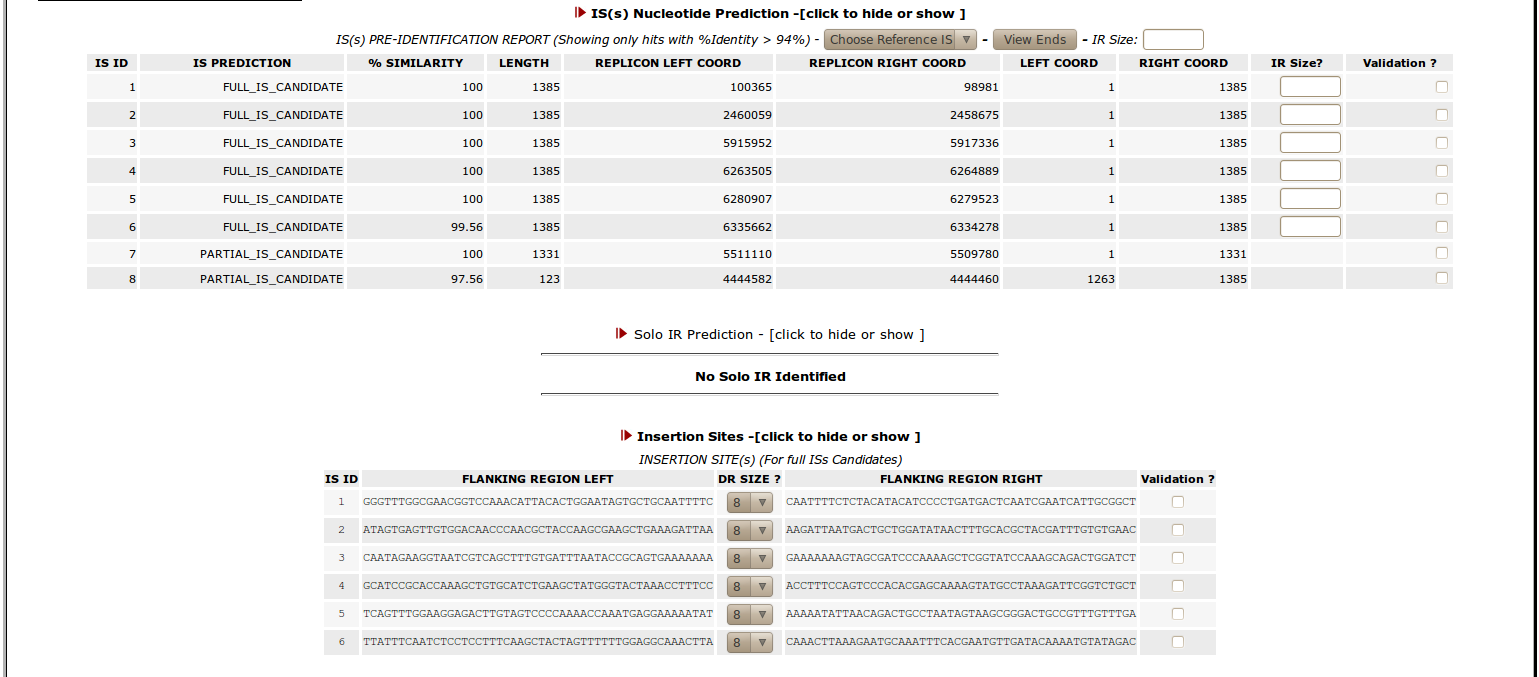
**

**Figure 4.4b**: Nucleotide sequence information

**
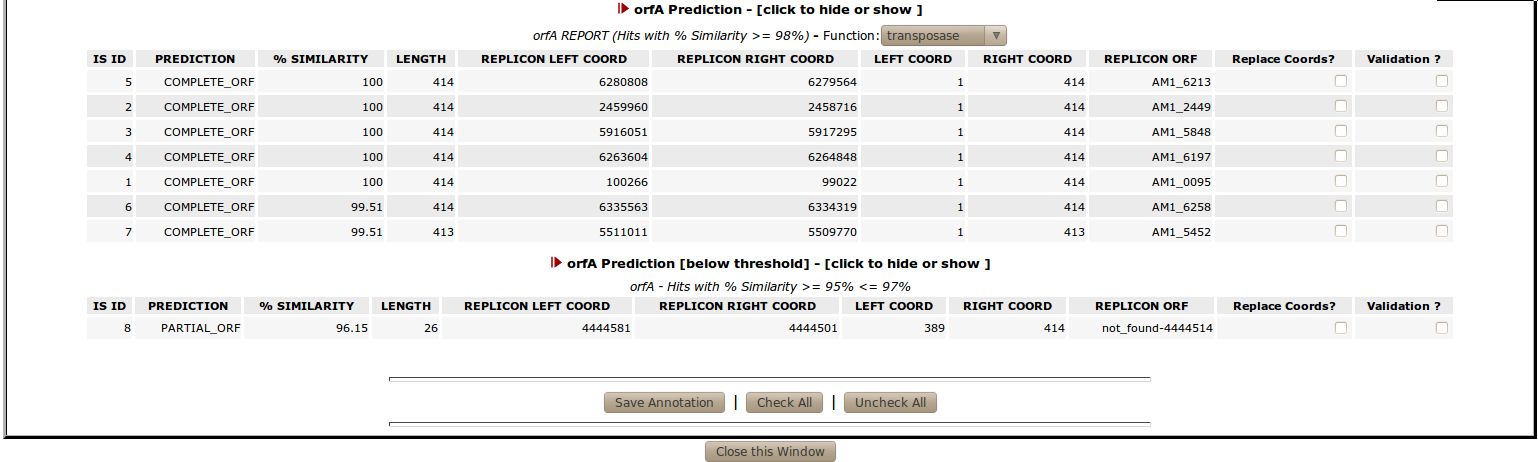
**

**Figure 4.4c**: Orf information

After validating the lines using the "check box" on the right, the option “Save Annotation” loads the validated information into the annotation table (figure 4.5a). This process could take some time to run and will generate a pop-up window with confirmation of the loading.

**a)**

**
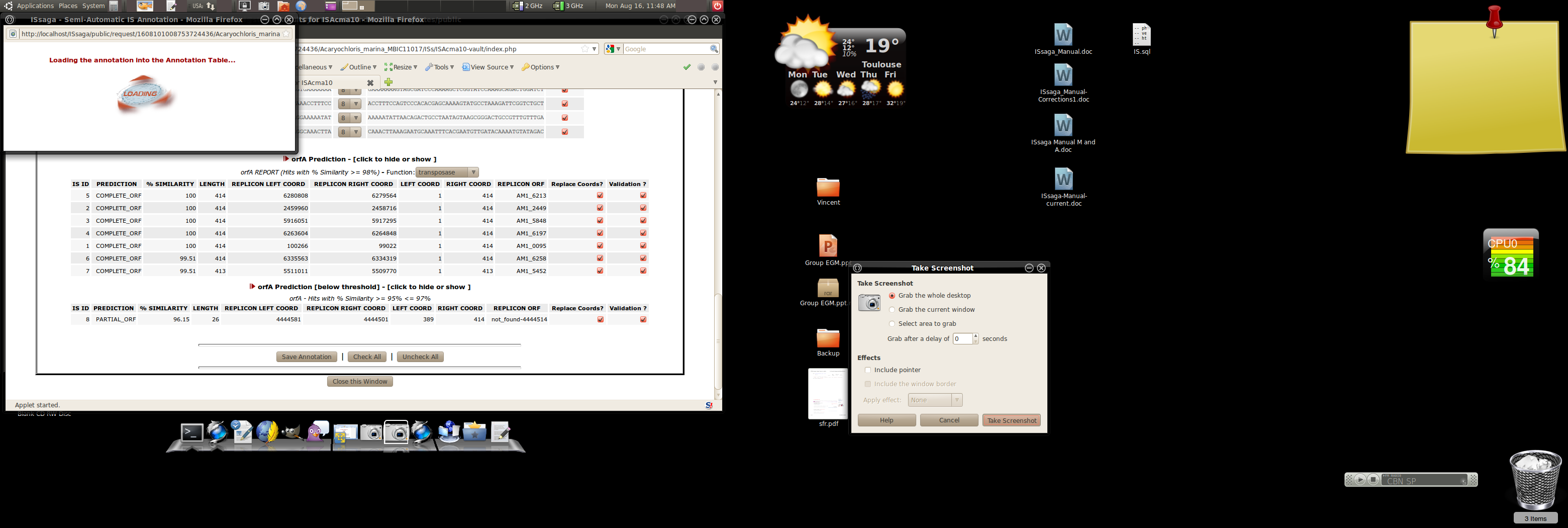
**

**b)**

**
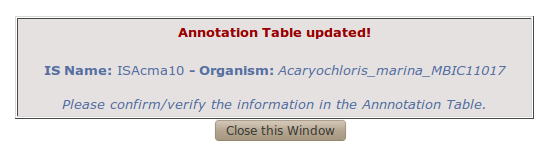
**

**Figure 4.5.** Loading the validated IS information into annotation table. **a**, loading screen. **b**, example of confirmation of the loading of the report into the annotation table.

**4.2. Reconstructing a disrupted IS.**

If there are partial IS candidates which could be disrupted by the product or action of another mobile genetic element, the system automatically generates the "Reconstruct Disrupted IS(s) Table" (figure 4.6) on the **IS Validation Report**. To reconstruct disrupted IS the user must set the option ***Reconstruct IS(s)*** to "*YES*", and provide the necessary annotation information in the fields: **ID**, **SLICE** and **Structure**. These options are mandatory in order for the system to reconstruct the IS. Once validated, the information is automatically loaded into the Annotation Table. Figure 4.6 shows an example of how to reconstruct a disrupted IS.

Reconstruct IS fields:

- *ID:* Unique identification number of each component of the disrupted IS.

- *SLICE:* Tags the IS fragments to be used in the reconstruction.

- *Structure:* C (complete reconstructed IS), P (partial reconstructed IS).

**a)**


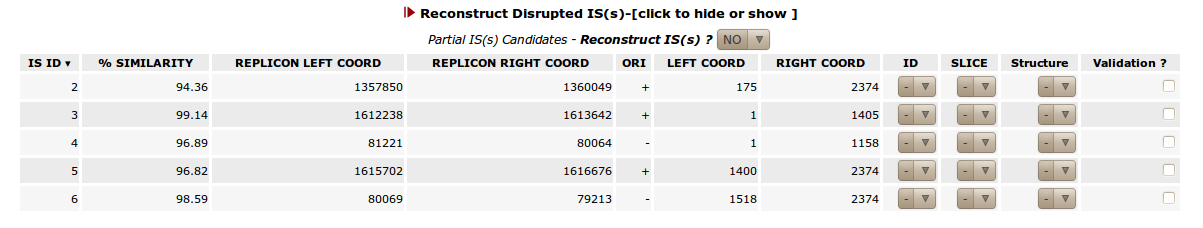


**b)**


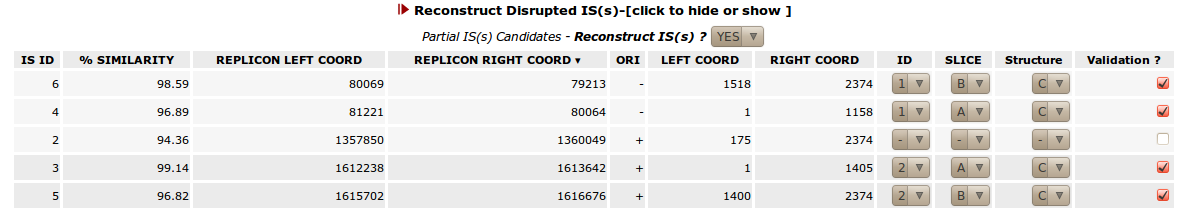


**Figure 4.6.** Example of reconstruction of disrupted ISs(example based on ISKol3, 2.374 bp). **a**,the empty table showing the partial IS candidates which could be disrupted. **b**, the reconstruction of two complete and disrupted ISs.

**Chapter 5.** **Manual Annotation Steps**

If an IS is not included in the ISfinder database, it is necessary to annotate a copy by hand and submit it to ISsaga so that it (and other genomic copies) will appear in the validation report. This copy is called the **reference copy**. This section describes the methods for identification and submission of the reference IS copy to ISsaga. All procedures are manual and require some annotation expertise.

The steps are the following:

- Use the *Pre-identified Different ISs* table to choose a region which could carry an IS.

- Identify and annotate the reference copy (using ISsaga tools).

- Request an IS name (using the ISfinder form) when you are sure that your ISs is complete. Only complete, non mutated sequences have a name in the ISfinder database.

- Identify and annotate the isoforms (protein sequence is more than **98% identical** and/or the DNA sequence is more than **95% identical)**, partials and solo IRs (semi-automatic using ISsaga).

- Submit the newly identified IS to ISfinder (using ISsaga or ISfinder website forms).

**5.1. Identification and annotation of the reference IS copy**

**- What is the reference IS copy?**

The reference IS copy is an IS stored in ISfinder. If a new IS identified by the user is not already included in ISfinder, it should be added manually as the reference copy by entering a set of information in a text file. This includes: **General information** (IS name, IS family); **Nucleotide sequence** **information** (IS nucleotide sequence with IRs and without DRs, Insertion sites, Length in bp); **Protein sequence information** (ORF sequence(s) and coordinates within the IS, ORF length in amino acids).

Figure 5.1 shows an example of a reference IS copy already submitted to ISfinder. To describe new ISs, the user must use the TOOLS link in the annotation page and follow the annotation protocol. We strong recommend the use of **IS notepad** (found in the drop-down menu in the **TOOLS**) to store the “draft” of the reference IS copy. The IS notepad provides a template file with all necessary fields required for ISfinder submission. For a new IS with more than one copy in the genome, a single representative example should be chosen.


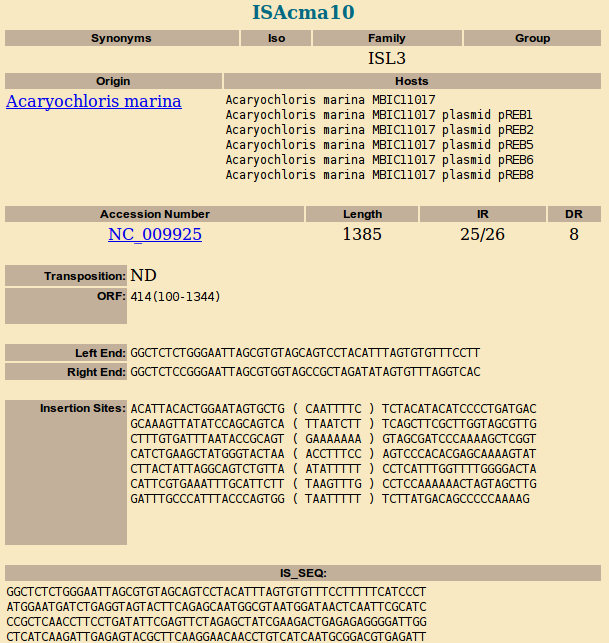


[Part of this picture has been deleted for brevity]


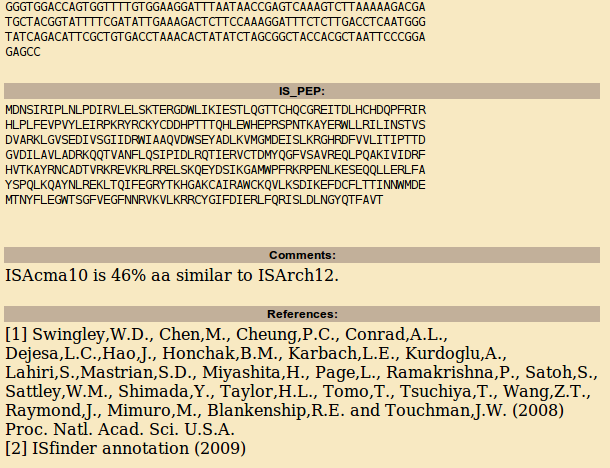


**Figure 5.1.** Example of an IS reference copy deposited in ISfinder database.

**- When is it necessary to identify and annotate a reference IS copy?**

If there are unfilled lines in the annotation table after the protein and automatic IS detection steps (Topic 4.1), these must be annotated by hand. In general, empty lines correspond to un-annotated IS elements (complete or partials). Figure 5.2 shows an example of an annotation table with annotated and unannotated lines. The orfs AM1_6225, AM1_6227, AM1_6237, AM1_6238, correspond to unannotated lines which must be annotated by hand, while AM1_6297 and AM1_6213 correspond to annotated ISAcma10.

**
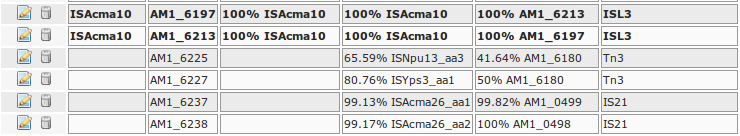
**

**Figure 5.2.** Example of annotation table showing annotated lines (in bold) and unannotated lines (Part of this picture has been deleted for brevity).

If all rows in the annotation are complete it is not necessary to use the manual annotation procedure. However, the manual annotation steps are normally required when dealing with new genomes. **Note:** Some unfilled lines in the annotation table could be false positives and the user should pay careful attention in each case, (Topic 5.3).

**5.1 How to obtain an IS name**

**- Determine whether the sequence is already registered under another name:**

- Perform a BLAST analysis against the entire ISfinder database (use BLASTp first) by simply pasting in the sequence (remove filter).

- If the protein sequence is more than **98% identical** and/or the DNA sequence is more than **95% identical** to an ISfinder entry, your sequence is an **isoform** and **does not require** a separate attribution. The name to use is that given in the database but send us a note of the bacterial species in which it was found.

- If the sequence is not in ISfinder but there are related sequences, this will indicate to which family the IS belongs.

- Fill in the online form: <http://www-is.biotoul.fr/is/is_name_attrib.html>(Figure 5.3).

- An email will be sent with the name usually within 5 working days.


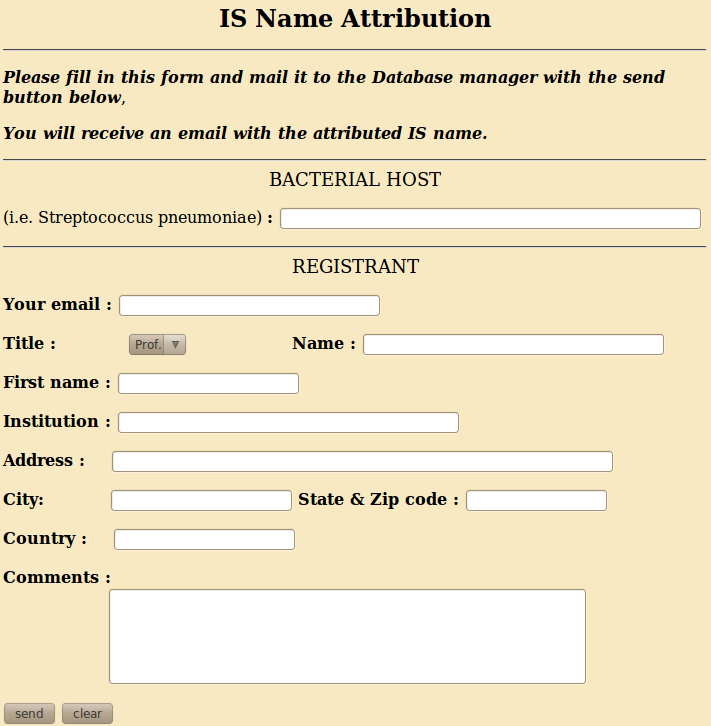


**Figure 5.3.** The IS Name attribution form. The user must complete all required fields.

**- How to annotate a new reference IS copy**

The procedure is the same for ISs with additional orfs (e.g. with passenger genes). However, the special case in which the Tpase is produced by translational frameshifting will be treated separately below.

- The use of the IS notepad is recommended to save each action made.

- Consult the table "MAJOR FEATURES OF PROKARYOTE IS FAMILIES" (<http://www-is.biotoul.fr/is/IS_infos/is_family.html> ) for guidance.

- Choose an IS to annotate. This appears as an un-annotated line in the annotation table. Generally the IS will appear on a single line if it includes a single orf, two successive lines if it includes two orfs etc. Examples of annotation of reference IS copies with a single or two orfs are shown in figure 5.4.


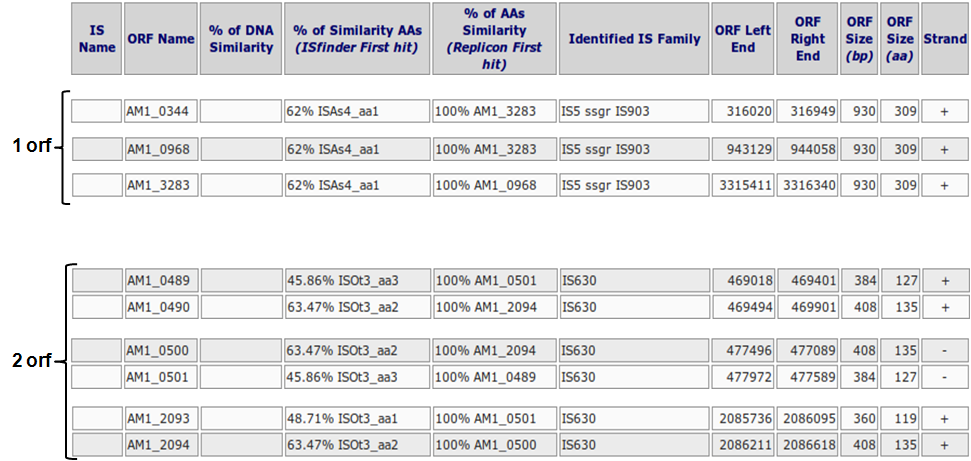


**Figure 5.4.** Annotation table showing an example of an IS with a single orf (three top line) and one with two orfs (bottom three lines).

- Identify and choose the DNA region which includes the IS with a nucleotide region which extends at least 800nt (depending on the IS family) on each side of the orf(s). It is important to note that the left end of the IS is defined as UPSTREAM of the transposase orf and is used to define the orientation of the IS in the genome.

In the example shown in the upper line of figure 5.4, the left orf end is at coordinate 316020. Extending this by 800nt gives the DNA coordinate 315220. Similarly, the right orf end is located at 316949 which can be extended to 317749. **Note:** For ISs located on the complementary strand, the extension must respect the orientation. In the example shown in figure 22 (bottom), the left end is at coordinate 477089. This should be extended to 476289, and the right end is at 477972, and should be extended to 478772.

- Use the Sequence Extractor (figure 5.5) to obtain the sequence:


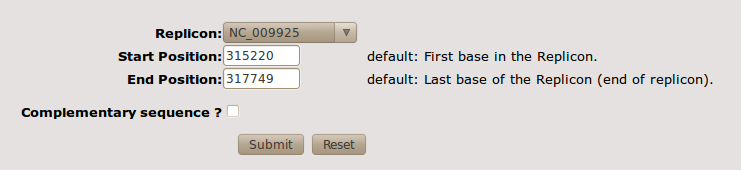


**Figure 5.5.** Sequence Extractor in the TOOLS drop-down menu

- BLAST the given sequence against the chromosome using the ***Replicon BLAST*** in the **TOOLS** drop-down menu to find any additional copies of the IS in the chromosome. If there is more than one copy of the IS, the BLAST result normally will show the beginning and end of the IS element. Ignore the first BLAST alignment (which will be the reference copy against itself) and look at the subsequent hits (figure 5.6). Note that if there is only one copy the user must find the correct ends of the given element by eye. In this step the user should consult the table of the major features of IS families to identify the correct types of end.


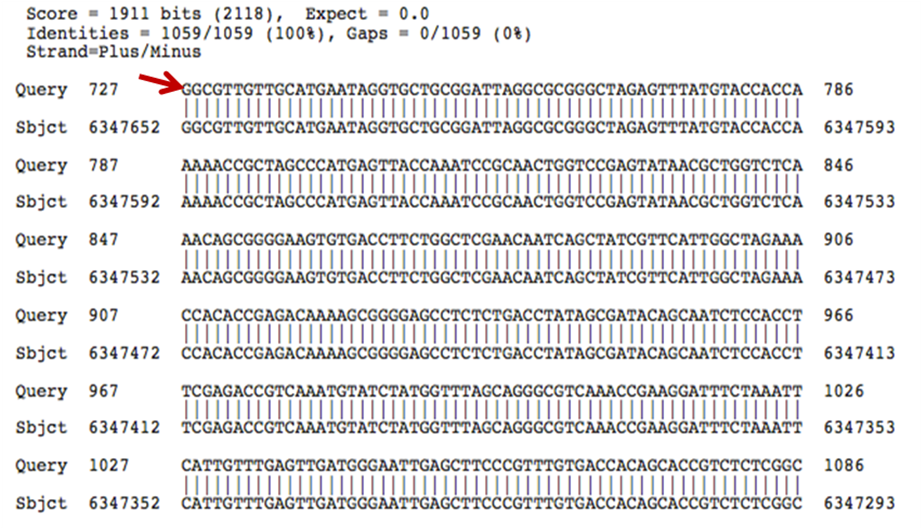


[Part of this picture has been deleted for brevity]

**
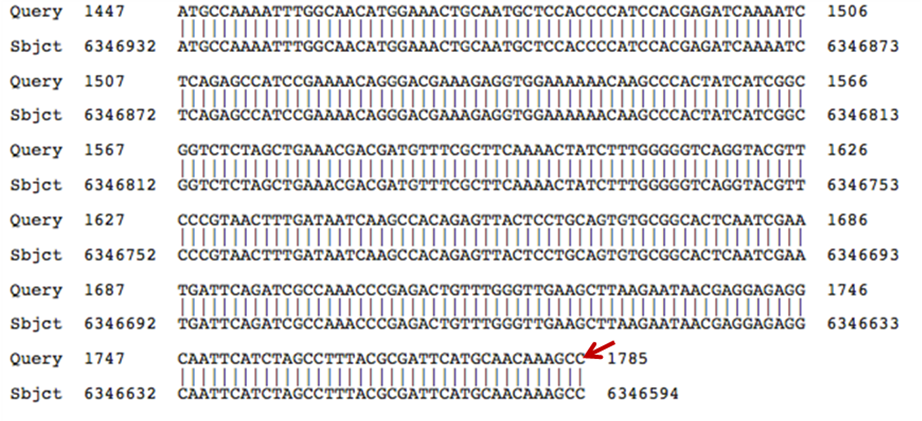
**

**Figure 5.6.** Example showing the beginning and end (red arrow) of the given IS element.

When there is only one copy of the given IS in the genome, the ***Replicon BLAST*** analysis will not provide the beginning and end of the element. For this, it is necessary to use the ***BLAST 2 Sequences*** (BLASTn the given sequence against itself, using the options without filter and increasing the e-value above 100. These settings are IMPORTANT since the default BLAST setting will not detect such small alignments). The result is shown in figure 5.7.


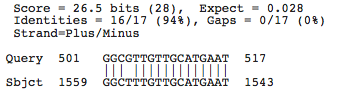


**Figure 5.7.** Example showing the inverted repeat.

- With the complete IS copy defined in this way it can now be extracted as a single entity with the correctly defined nucleotide ends using the Sequence Extractor again (figure 23).

- The next step will be to define the terminal inverted repeats (IR) for those ISs which include such sequences (IS*200*/IS*605*, IS*110* and IS*91* family members do not include IRs). This type of information is included in the "MAJOR FEATURES OF PROKARYOTE IS FAMILIES" table. The ***View Ends*** function (figure 5.8 top) should be used for defining terminal IRs. This must be done manually as shown in the bottom part of the figure. In this case the IRs are 17nts/18nts (figure 5.8 bottom).


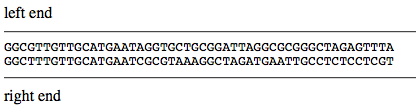


GGCGTTGTTGCATGAATAGGTGCTGCGGATTAGGCGCGGGCTAGAGTTTA
GGCTTTGTTGCATGAATCGCGTAAAGGCTAGATGAATTGCCTCTCCTCGT

**Figure 5.8.** The view ends function and the manual IR definition.

- For those IS which generate flanking target repeats on insertion (DR) these can be identified ***Predictor & Flank Extractor*** in the **SEMI-AUTOMATIC** drop-down menu (figure 5.9 top). This value is generated automatically but should be verified by eye. The table also shows the number of copies of the IS found in the replicon. **Note**: this table may give information concerning IS-mediated genome rearrangements. In the case of an IS in which members give a DR of defined length, examples may be observed which do not have flanking DRs but in which the flanking sequence at one end of the IS can be found at the end of a second copy in the genome. This implies that recombination has occurred between the two ISs in question to generate, for example, an inversion.

- After verification, the “Extract DRs” button will generate the DR nucleotide sequences formatted for submission to ISfinder (figure 5.9 bottom).


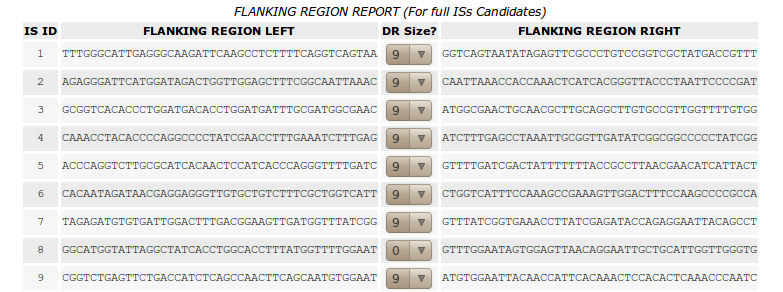


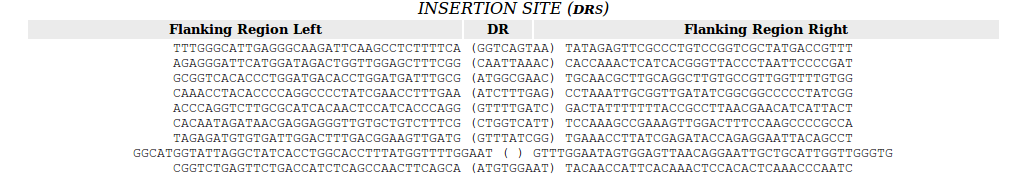


**Figure 5.9.** Predictor & Flank Extractor

- It is then necessary to identify the IS proteins and verify whether the previous annotation (e.g. the original files which were submitted for annotation) is correct. In this step the user must determine by eye if the start and stop codon of each IS orf are correctly annotated. ISsaga provides the necessary tools to do this (***ORF Finder***, ***Filter DNA***, ***Revert DNA***, and ***Translator*** in **TOOLS** the drop-down menu).

**- The IS Notepad: example of the fields required for submission of the reference IS**

The different fields included in the template provided in IS Notepad are described below for the example of IS*Acma6, a* 1059 bp member of the IS*5* family subgroup IS*903*. (upper line of figure 22)

1) The Genome Fragment carrying the IS.

The DRs are in bold and colored in red, the ends of the IS are underlined, and the transposase orf is colored in orange.

**ISAcma6 – Family IS*5* ssgr IS*903***

1 tatcgtcaat gtcctcaagc ctattatttc aagcatgagc ggcggttaac ggtccccacg

61 gctttcggtt cacccacctt tggtaaagca atccatcggg ctttggccca aatttaccaa

121 gactggtcct atgccgaccc cattccgccc ctgcaatggt ttgccgactg ttggcaacag

181 catatcggcg aactcagtga gtcccaaatt catgaaggct ggcaagccct ggagacttac

241 tatcgccaat atgtggctcc tcaatcgatg ttgcgaaaac ctttgggcat tgagggcaag

301 attcaagcct cttttca**ggt cagtaa**ggcg ttgttgcatg aataggtgct gcggattagg

361 cgcgggctag agtttatgta ccaccaaaaa ccgctagccc atgagttacc aaatccgcaa

421 ctggtccgag tataacgctg gtctcaaaca gcggggaagt gtgaccttct ggctcgaaca

481 atcagctatc gttcattggc tagaaaccac accgagacaa aagcggggag cctctctgac

541 ctatagcgat acagcaatct ccaccttcga gaccgtcaaa tgtatctatg gtttagcagg

601 gcgtcaaacc gaaggatttc taaattcatt gtttgagttg atgggaattg agcttcccgt

661 ttgtgaccac agcaccgtct ctcggcggaa aggtcagcta tcgatttcat tgcccgttat

721 acctaagcaa ggtgccattc acgtcgttat cgatagcaca gggatcaaag tctatggtga

781 aggagagtgg aaaaccaggc aacatggtgt gagcaaacgt cggacgtggc gtaaacttca

841 cttgagtgtt gatgagtcca ctggagaaat tctcacgggt gtcgtgacaa ccaatgacgt

901 tcaggatggt gaagtgtttg aagatctcct tgaaggcatc gacgatgaga ttgaacaggt

961 ctctgctgac ggagcctatg accaaagtca ttgctatgat gccctcatgg agcgcaatgc

1021 cacagctgca attcccccaa ggaagaatgc caaaatttgg caacatggaa actgcaatgc

1081 tccaccccat ccacgagatc aaaatctcag agccatccga aaacagggac gaaagaggtg

1141 gaaaaaacaa gcccactatc atcggcggtc tctagctgaa acgacgatgt ttcgcttcaa

1201 aactatcttt gggggtcagg tacgttcccg taactttgat aatcaagcca cagagttact

1261 cctgcagtgt gcggcactca atcgaatgat tcagatcgcc aaacccgaga ctgtttgggt

1321 tgaagcttaa gaataacgag gagaggcaat tcatctagcc tttacgcgat tcatgcaaca

1381 aagcc**ggtca gtaa**tataga gttcgccctg tccggtcgct atgaccgttt agactggcta

1441 gacgatggcc tagaactgat tgactacaaa accagtaaaa ctgttaagcc cccagaagcc

1501 attgatgtgc agttggggct ttactattta gcgctggaac agacttatca ccatgccctt

1561 aaacgcttga gcttgatcta tattacgcag caatcaatgc atttcttatg aggttactcc

1621 cgatcattta gaccaaatca aaggtttgat tggcgactta gcactaaaac tgaggtctga

1681 tcaagactgg catctgaagt gacccccaag attcggacaa gtccttaagc

2) Transposase length in amino acids and coordinates in the IS

310 aa (75-1004)

MSYQIRNWSEYNAGLKQRGSVTFWLEQSAIVHWLETTPRQKRGASLTYSDTAISTFETVKCIYGLAGRQTEGFLNSLFELMGIELPVCDHSTVSRRKGQLSISLPVIPKQGAIHVVIDSTGIKVYGEGEWKTRQHGVSKRRTWRKLHLSVDESTGEILTGVVTTNDVQDGEVFEDLLEGIDDEIEQVSADGAYDQSHCYDALMERNATAAIPPRKNAKIWQHGNCNAPPHPRDQNLRAIRKQGRKRWKKQAHYHRRSLAETTMFRFKTIFGGQVRSRNFDNQATELLLQCAALNRMIQIAKPETVWVEA

3) Terminal inverted repeats (IRs)

**IR: 18/20**

**LEFT END :** GGCGTTGTTGCATGAATAGGTGCTGCGGATTAGGCGCGGGCTAGAGTTTA

**RIGHT END:** GGCTTTGTTGCATGAATCGCGTAAAGGCTAGATGAATTGCCTCTCCTCGT

4) Flanking regions with the flanking direct target repeats (DR)

**INSERTION SITE: 9 bp**

GAGGGCAAGATTCAAGCCTCTTTTCA (GGTCAGTAA) TATAGAGTTCGCCCTGTCCGGTCGC

ATGGATAGACTGGTTGGAGCTTTCGG (CAATTAAAC) CACCAAACTCATCACGGGTTACCCT

CCTGGATGACACCTGGATGATTTGCG (ATGGCGAAC) TGCAACGCTTGCAGGCTTGTGCCGT

CCCCAGGCCCCTATCGAACCTTTGAA (ATCTTTGAG) CCTAAATTGCGGTTGATATCGGCGG

TGCGCATCACAACTCCATCACCCAGG (GTTTTGATC) GACTATTTTTTTACCGCCTTAACGA

AACGAGGAGGGTTGTGCTGTCTTTCG (CTGGTCATT) TCCAAAGCCGAAAGTTGGACTTTCC

TGATTGGACTTTGACGGAAGTTGATG (GTTTATCGG) TGAAACCTTATCGAGATACCAGAGG

TATCACCTGGCACCTTTATGGTTTTGGAAT ( ) GTTTGGAATAGTGGAGTTAACAGGAATTG

TCTGACCATCTCAGCCAACTTCAGCA (ATGTGGAAT) TACAACCATTCACAAACTCCACACT

5) Nucleotide Sequence

**IS Sequence: 1059** **bp**

GGCGTTGTTGCATGAATAGGTGCTGCGGATTAGGCGCGGGCTAGAGTTTATGTACCACCAAAAACCGCTAGCCCATGAGTTACCAAATCCGCAACTGGTCCGAGTATAACGCTGGTCTCAAACAGCGGGGAAGTGTGACCTTCTGGCTCGAACAATCAGCTATCGTTCATTGGCTAGAAACCACACCGAGACAAAAGCGGGGAGCCTCTCTGACCTATAGCGATACAGCAATCTCCACCTTCGAGACCGTCAAATGTATCTATGGTTTAGCAGGGCGTCAAACCGAAGGATTTCTAAATTCATTGTTTGAGTTGATGGGAATTGAGCTTCCCGTTTGTGACCACAGCACCGTCTCTCGGCGGAAAGGTCAGCTATCGATTTCATTGCCCGTTATACCTAAGCAAGGTGCCATTCACGTCGTTATCGATAGCACAGGGATCAAAGTCTATGGTGAAGGAGAGTGGAAAACCAGGCAACATGGTGTGAGCAAACGTCGGACGTGGCGTAAACTTCACTTGAGTGTTGATGAGTCCACTGGAGAAATTCTCACGGGTGTCGTGACAACCAATGACGTTCAGGATGGTGAAGTGTTTGAAGATCTCCTTGAAGGCATCGACGATGAGATTGAACAGGTCTCTGCTGACGGAGCCTATGACCAAAGTCATTGCTATGATGCCCTCATGGAGCGCAATGCCACAGCTGCAATTCCCCCAAGGAAGAATGCCAAAATTTGGCAACATGGAAACTGCAATGCTCCACCCCATCCACGAGATCAAAATCTCAGAGCCATCCGAAAACAGGGACGAAAGAGGTGGAAAAAACAAGCCCACTATCATCGGCGGTCTCTAGCTGAAACGACGATGTTTCGCTTCAAAACTATCTTTGGGGGTCAGGTACGTTCCCGTAACTTTGATAATCAAGCCACAGAGTTACTCCTGCAGTGTGCGGCACTCAATCGAATGATTCAGATCGCCAAACCCGAGACTGTTTGGGTTGAAGCTTAAGAATAACGAGGAGAGGCAATTCATCTAGCCTTTACGCGATTCATGCAACAAAGCC

**Note**: This notepad example is valid for most known IS types. For particular cases, or when ends are difficult to identify, please contact ISfinder team.

**- Frameshifting: a special example**

ISs belonging to families IS*1*, IS*3*, IS*5* ssgr IS*427*, and some members of IS*630* carry two consecutive orfs, A and B, but the transposase AB is a fusion product produced by a programmed -1 translational frameshift from both orfs. ISsaga will identify the A frame and also part of the B frame (depending on the previous annotation). In these cases it is necessary to identify the AB frameshift product. An example is presented in figure 5.4 (bottom). All steps followed for an IS with a single orf should be followed. This should generate two orfs as shown below.

**ISAcma31 – orfA (54-413) 119**

MPAPYSYDLRRKAVDAFKNGERKVDICRMLNISRNTLHLWIVREEATGDCQAITNYQQGARHKITDWERF

REFAQEHGGKTQAQMAKLWGDNVTQQNISDALRKLGLSRKKDLWLSRTR

**ISAcma31 – orfB (529-936) 135**

MGQRFYDLKSGKRTERVSFIAALKEGQLFSPMTFEGSCNRLLFEAWLQQSLISQLQLGDVIVIDNASFHHGQRIEEIVAEAGCEIWYLPSYSPDLNKIERWWFVLKNWMKQRWDEFDTFRDCVDAAFKERTNIYP

In general, the second frame will be truncated and not include the correct N-terminus. A three frame translation should be performed using ***Translator*** in the **TOOLS** drop-down menu as shown below.

**Translation**

1 CGAAACTATGTTAGTACTATTGATATAGAAGATATCCCTACATTAGTATGCCAGCACCCT

1 R N Y V S T I D I E D I P T L V C Q H P

1 E T M L V L L I * K I S L H * Y A S T L

1 K L C * Y Y * Y R R Y P Y I S M P A P

61 ATAGTTATGACCTTCGTCGTAAAGCCGTTGATGCCTTCAAGAATGGTGAACGGAAAGTCG

21 I V M T F V V K P L M P S R M V N G K S

21 * L * P S S * S R * C L Q E W * T E S R

20 Y S Y D L R R K A V D A F K N G E R K V

121 ATATCTGTCGAATGTTGAACATCAGCCGTAATACCTTGCATCTATGGATAGTCCGCGAAG

41 I S V E C * T S A V I P C I Y G * S A K

41 Y L S N V E H Q P * Y L A S M D S P R R

40 D I C R M L N I S R N T L H L W I V R E

181 AAGCTACAGGTGACTGTCAGGCGATTACCAACTACCAACAAGGGGCTCGCCACAAGATCA

61 K L Q V T V R R L P T T N K G L A T R S

61 S Y R * L S G D Y Q L P T R G S P Q D H

60 E A T G D C Q A I T N Y Q Q G A R H K I

241 CAGATTGGGAGCGCTTTCGTGAATTTGCTCAAGAGCATGGGGGTAAAACCCAGGCCCAAA

81 Q I G S A F V N L L K S M G V K P R P K

81 R L G A L S * I C S R A W G * N P G P N

80 T D W E R F R E F A Q E H G G K T Q A Q

301 TGGCTAAGCTATGGGGGGATAATGTCACCCAGCAAAATATCAGTGATGCCTTGAGAAAAC

101 W L S Y G G I M S P S K I S V M P * E N

101 G * A M G G * C H P A K Y Q * C L E K T

100 M A K L W G D N V T Q Q N I S D A L R K

361 TGGGATTGAGTCG**AAAAAAA**GACCTATGGCTATCGAGAACGAGATGAAACACAACGTCAA

121 W D *  **V E K K T Y G Y R E R D E T Q R Q**

121 G I E S K K R P M A I E N E M K H N V K

120 L G L S R K K D L W L S R T R * N T T S

421 GCATTTATAGAACGATTACAGACCAAACACCCTCATCAGATCGTCTATGTAGACGAAGCG

141  **A F I E R L Q T K H P H Q I V Y V D E A**

141 H L * N D Y R P N T L I R S S M * T K R

140 S I Y R T I T D Q T P S S D R L C R R S

481 GGTATCGATAATCGAGCGGACTATCCTTATGGATACTGCCCTGTGGGTCAACGATTTTAT

161  **G I D N R A D Y P Y G Y C P V** G Q R F Y

161 V S I I E R T I L M D T A L W V N D F M

160 G Y R * S S G L S L W I L P C G S T I L

541 GACCTCAAATCAGGCAAGCGGACAGAGCGAGTCAGTTTTATTGCTGCGCTCAAAGAAGGC

181 D L K S G K R T E R V S F I A A L K E G

181 T S N Q A S G Q S E S V L L L R S K K A

180 * P Q I R Q A D R A S Q F Y C C A Q R R

601 CAGTTATTTTCTCCCATGACCTTTGAAGGGTCTTGCAATCGGTTGTTATTCGAAGCGTGG

201 Q L F S P M T F E G S C N R L L F E A W

201 S Y F L P * P L K G L A I G C Y S K R G

200 P V I F S H D L * R V L Q S V V I R S V

661 TTACAGCAGAGTCTTATATCCCAGTTACAACTTGGCGATGTGATTGTGATTGATAATGCC

221 L Q Q S L I S Q L Q L G D V I V I D N A

221 Y S R V L Y P S Y N L A M * L * L I M P

220 V T A E S Y I P V T T W R C D C D * * C

721 AGTTTTCATCATGGCCAGAGGATCGAAGAAATCGTAGCTGAAGCAGGCTGTGAGATTTGG

241 S F H H G Q R I E E I V A E A G C E I W

241 V F I M A R G S K K S * L K Q A V R F G

240 Q F S S W P E D R R N R S * S R L * D L

781 TATCTACCGAGCTATTCTCCAGATCTCAATAAAATCGAGCGATGGTGGTTTGTGCTCAAA

261 Y L P S Y S P D L N K I E R W W F V L K

261 I Y R A I L Q I S I K S S D G G L C S K

260 V S T E L F S R S Q * N R A M V V C A Q

841 AACTGGATGAAGCAGCGGTGGGATGAATTTGATACCTTTCGTGATTGTGTGGATGCTGCT

281 N W M K Q R W D E F D T F R D C V D A A

281 T G * S S G G M N L I P F V I V W M L L

280 K L D E A A V G * I * Y L S * L C G C C

901 TTCAAAGAGCGTACTAACATATATCCG

301 F K E R T N I Y P

301 S K S V L T Y I

300 F Q R A Y * H I S

The A frame is indicated in red and the B frame in reading phase -1 compared to A is shown in green. The correct B frame is defined by identifying the first termination codon in the B reading phase located within the A frame (in this example this is TGA, codon 123) giving a B frame modified at its N-terminus.

**ISAcma31 – orfB (376-936) 186**

**VEKKTYGYRERDETQRQAFIERLQTKHPHQIVYVDEAGIDNRADYPYGYCPV**GQRFYDLKSGKRTERVSFIAALKEGQLFSPMTFEGSCNRLLFEAWLQQSLISQLQLGDVIVIDNASFHHGQRIEEIVAEAGCEIWYLPSYSPDLNKIERWWFVLKNWMKQRWDEFDTFRDCVDAAFKERTNIYP

To obtain the true frameshifted AB frame some knowledge of the frameshift motif and frameshifting rules is necessary. Typical frameshifting motifs are shown in the table 5.1. The most common involve A7 or A6G. In this case, the relevant sequence is probably AAAAAAA at position 374-380 (bold and underlined in the example). The corresponding amino acid sequence for the frameshifted product is presented below.

**ISAcma31 – orfAB (54-936) 293**

MPAPYSYDLRRKAVDAFKNGERKVDICRMLNISRNTLHLWIVREEATGDCQAITNYQQGARHKITDWERFREFAQEHGGKTQAQMAKLWGDNVTQQNISDALRKLGLSRK**KTYGYRERDETQRQAFIERLQTKHPHQIVYVDEAGIDNRADYPYGYCPV**GQRFYDLKSGKRTERVSFIAALKEGQLFSPMTFEGSCNRLLFEAWLQQSLISQLQLGDVIVIDNASFHHGQRIEEIVAEAGCEIWYLPSYSPDLNKIERWWFVLKNWMKQRWDEFDTFRDCVDAAFKERTNIYP

| **Occurence in the IS3 family** | **Motif (X-XXZ-ZZN) / (Z-ZZN)** |
| --- | --- |
| 140  80  11  8  7  7  7  5  3  2  1  1  1  27  16  13  12  2 | A-AAA-AAG  A-AAA-AAA  G-GGA-AAG  G-GGA-AAC  A-AAA-AAC  U-UUA-AAG  U-UUU-UUC  U-UUA-AAA  G-GGA-AAA  U-UUU-UUG  U-UUU-UUU  C-CCA-AAA  C-CCA-AAG  A-AAG  U-UUC  A-AAA  U-UUU  G-AAG |

**Table 5.1.** Frameshift motifs. (Modified from Fayet and Prère, Nucleic Acids and Molecular Biology 24, Chapter 12, John F. Atkins and Raymond F. Gesteland 2009 ). Codons are shown in the pre-shifted frame.

**- Partial ISs**

Many genomes carry partial IS copies, but without the corresponding complete copy (reference IS copy). In these cases it is necessary to include the name "partial" in the IS NAME field and complete all required partial fields (*IS Left End* ,*IS Right End,* *IS Coordinates Present* and *Length of Partial*) in the Annotation Table (figure 5.10). Note: more details about partial IS, can be found in **Annotation examples.**


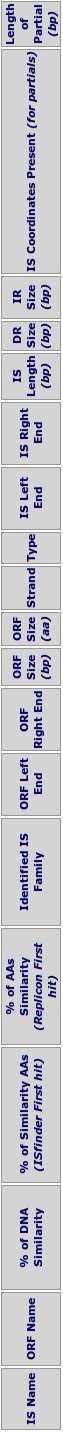

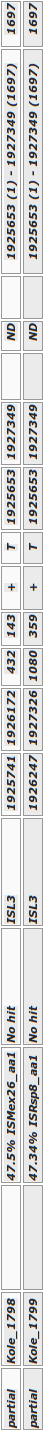


**Figure 5.10.** Example of partial IS in the Annotation Table.

**5.3 Dealing with false positives**

Some unannotated lines in the **Annotation Table** could be result of a false positive prediction and must be treated manually. The prediction & estimation results in the **Annotation page** uses the rules shown in figure 5.11, but all predicted IS-associated orfs will appear in the **Annotation Table**, to avoid exclusion of all regions which could contain an IS. The final decision as to whether the prediction is a false-positive or a true IS must be decided by the annotator. The majority of false-positives belong to the IS*21* (accessory gene helper of transposition), IS*91* and Tn*3* (accessory gene, similar to integrases or resolvases) and IS*66* (orfA) families. The rules presented in figure 28 must be used to remove the false positives. In other circumstances false positives from other families might appear. They generally appear with a low identity score with the closest ISfinder protein. In case of doubts please contact the ISfinder team.

**
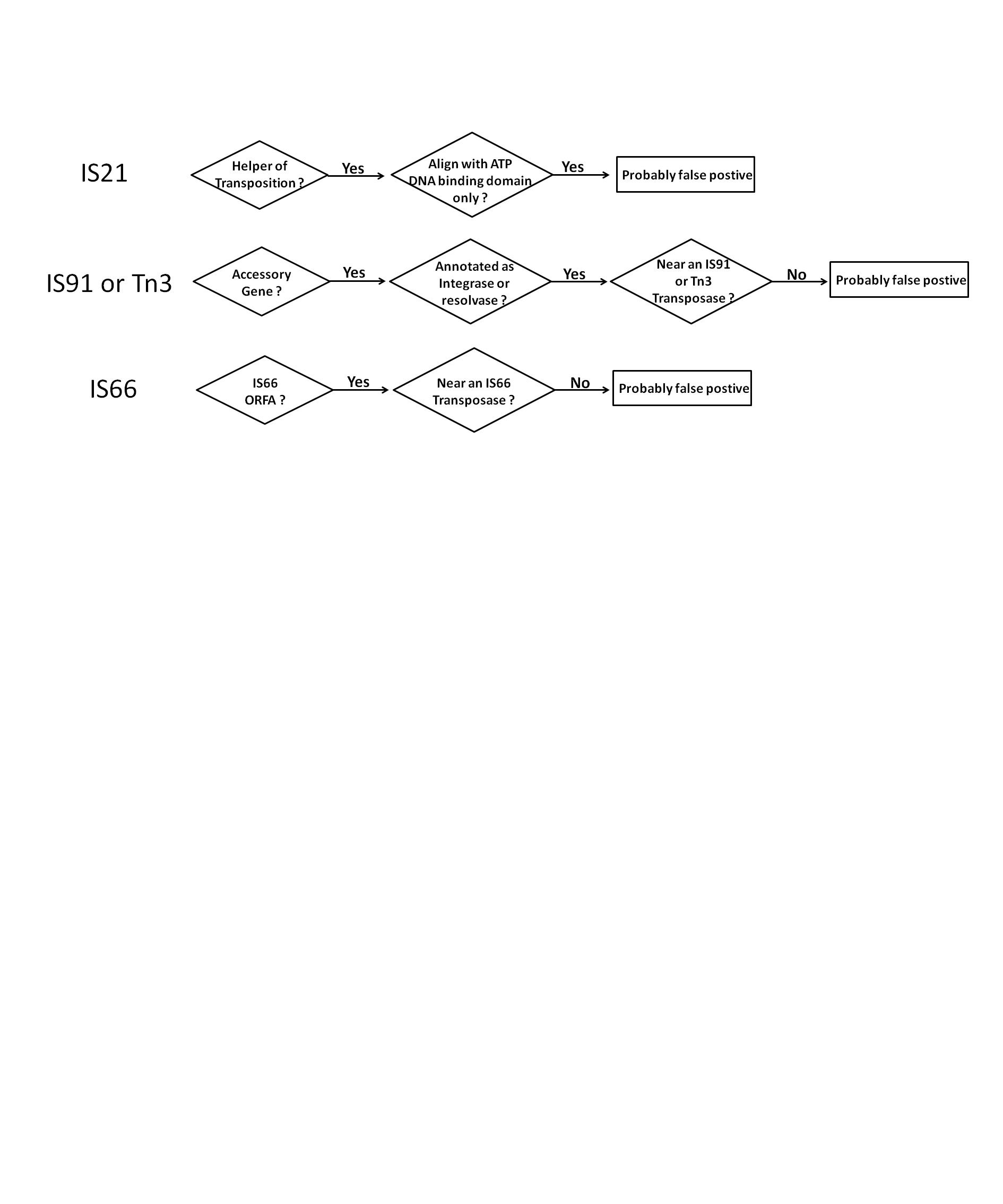
**

**Figure 5.11.** Rules to determine false positives in the Annotation Table.

**5.4 Studying the impact and role of IS using the IS ORFs Context Table**

The study and analysis of the role of the annotated ISs in orf disruption or inactivation is not a mandatory step, but is recommended since it provides an idea of how the ISs have impacted the host genome. The ORF Context Table (figure 5.12) is based on the **Annotation Table**, and includes information concerning the genes flanking each identified IS-associated orf. This will also provide information indicating whether the IS has disrupted a target gene. This table, like the **Annotation Table**, is also dynamically generated and fully clickable.

**
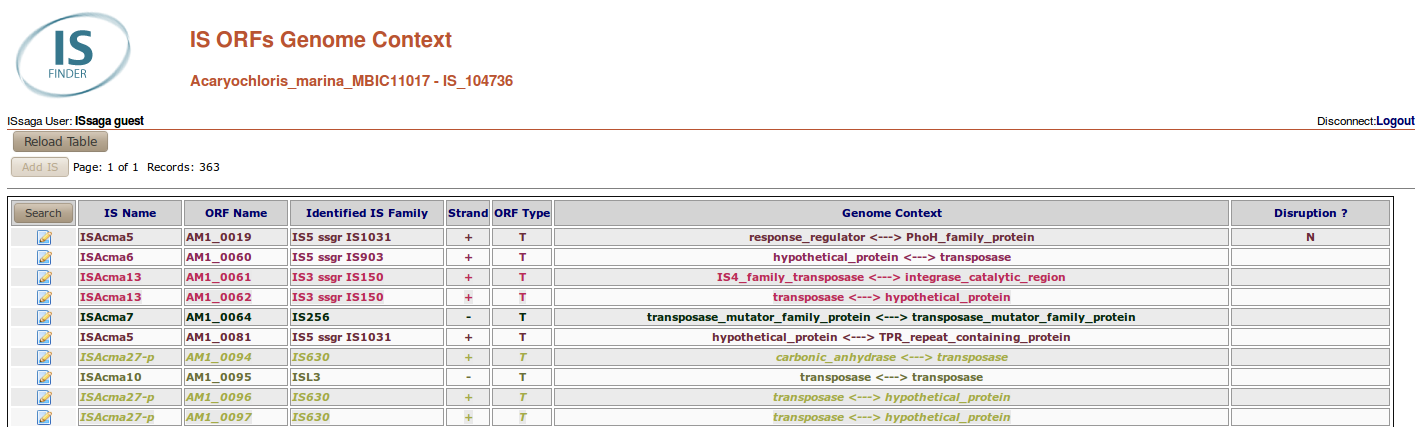
**

[Part of this picture has been deleted for brevity]


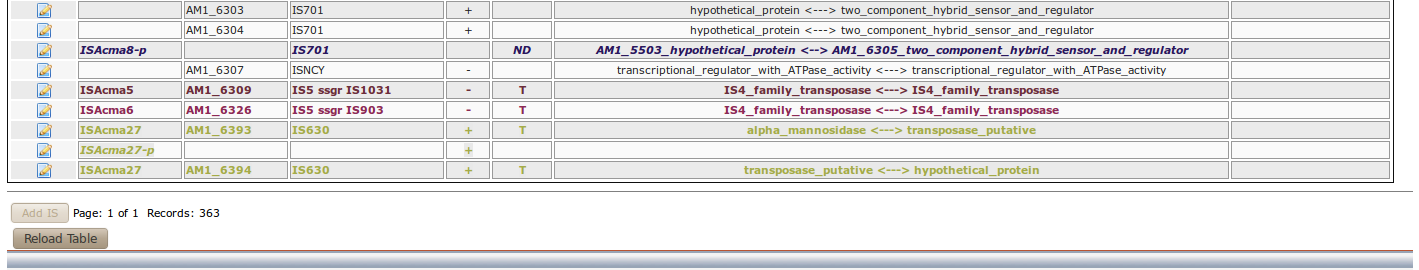


**Figure 5.12.** ORF Context Table displaying the information related to the annotation and the putative content of each flanking region.

The **IS ORF Context Table** is composed of five fields provided by the **Annotation Table** (IS Name, ORF Name, Identified IS Family, Strand, Type) in addition to two specific fields:

- Genome Context: This shows the flanking orf content

- Disruption: Shows whether the annotated IS is involved in disruption or inactivation of the orfs present near the insertion site.

Clicking on any other field containing information will open an editing page with more detailed information for the given line (figure 5.12). This can be used to edit information and can also be accessed from the left-most column of the table.

**5.4.3. Editing the IS ORF Context Table**

The editing page (figure 5.13) includes all headings listed above together with an additional four headings (IS Structure, IS Left End, IS Right End, IS Length) provided by the **Annotation Table**.

- Genome Context: provides information on the function of the flanking orfs

- Disruption: Provides information indicating whether the IS interrupts a target orf

- Disruption Comments: Space for including observations regarding annotation and identification of disruptions.

- Upstream Border: 1000bp sequence upstream of the IS-associated orf.

- Downstream Border: 1000bp sequence downstream of the IS-associated orf.

All other headings in this table are ‘read only’.


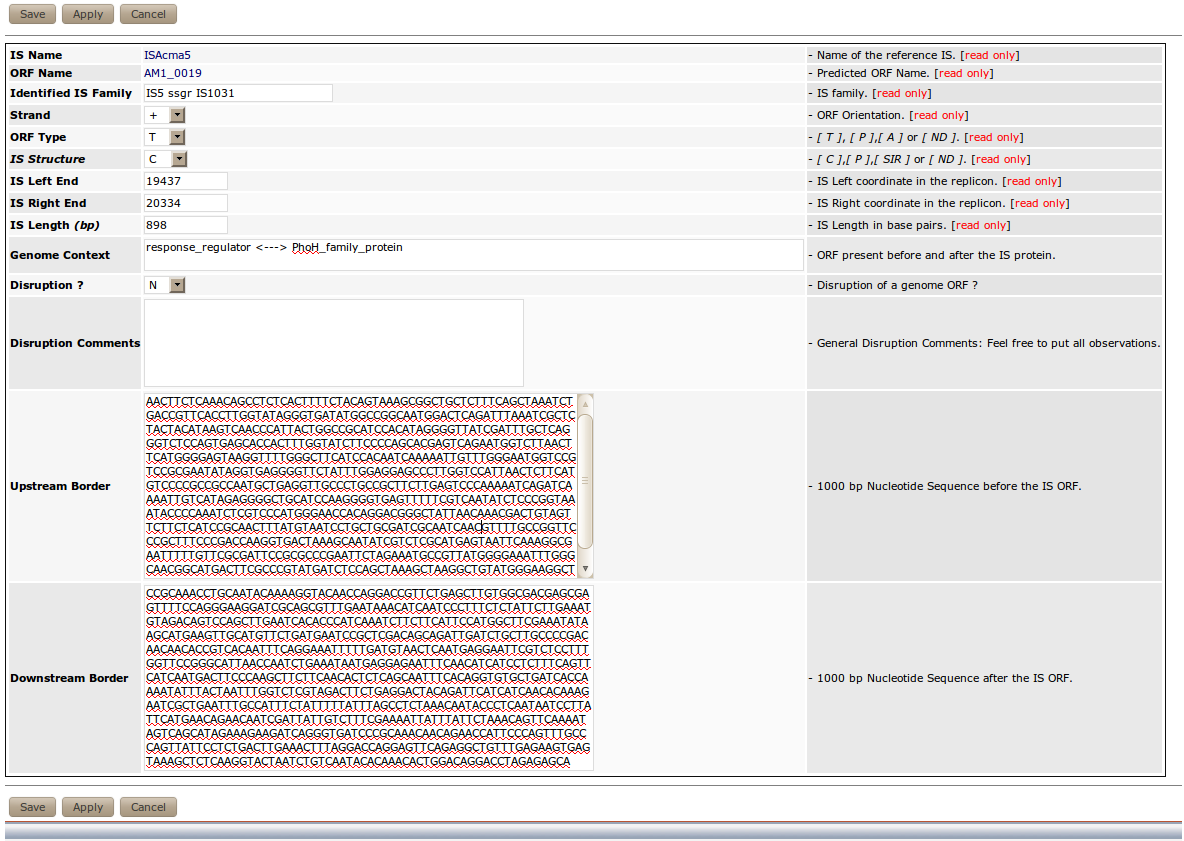


**Figure 5.13.** IS ORF Context Table with editable fields.

**5.4 Submitting a newly identified IS to ISfinder**

After finishing and verifying the annotation, the user should submit newly identified ISs to ISfinder using the the link Validation "Submit IS to ISfinder" (figure 5.14). ISfinder does not accept partial IS copies. It is therefore not necessary to submit these to ISfinder.

**We rely on you to enrich ISfinder by submitting your own sequences. Please help.**

- - Before submitting, make sure you have a registered IS name
  - Fill in the online form
  - Newly submitted ISs are added to the public database about once a week.

You may also ask us to withhold your IS from the public database until you wish it to be released (but please do not forget to recontact us). All sequences will be transferred to the public database after a period of six months from submission.

**
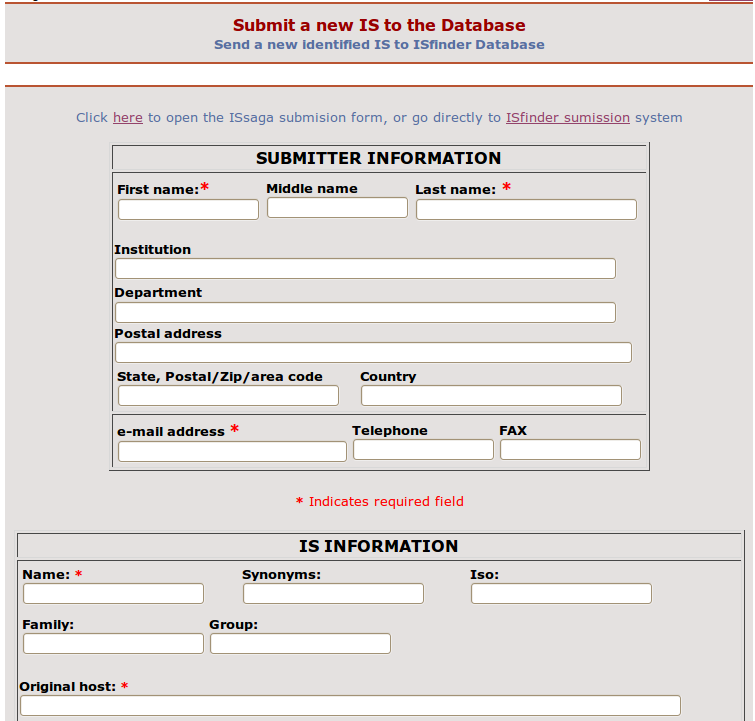
**

**Figure 5.14.** Example of the IS submission form (Part of this picture has been deleted for brevity).

**Chapter 6.** **Finishing the annotation**

Finishing the annotation is the last step of the ISsaga process. When there are no remaining un-annotated lines in the annotation table and all transposase, complete and partial IS have been verified, the user can finish the annotation and send the results for validation by the ISfinder team. After clicking on ***Finish the Annotation***, the user will no longer have access to the annotation in ISsaga until it has been validated. It is therefore **important** that the newly annotated genome be downloaded as an Excel or a new GenBank file prior to "finishing". All annotation made with ISsaga goes through a rigorous quality control.

**6.1 Exporting the annotation to a new GenBank file**

If the user wishes to analyze their results using other bioinformatics tools, it is possible to extract the annotation in a new GenBank file with all the correct information and tags related to the IS annotation. The user can also extract the annotation results in an Excel file format. To do this, use the link Annotation "Extract Annotation".

The figure 6.1 shows an example of GenBank file extraction.


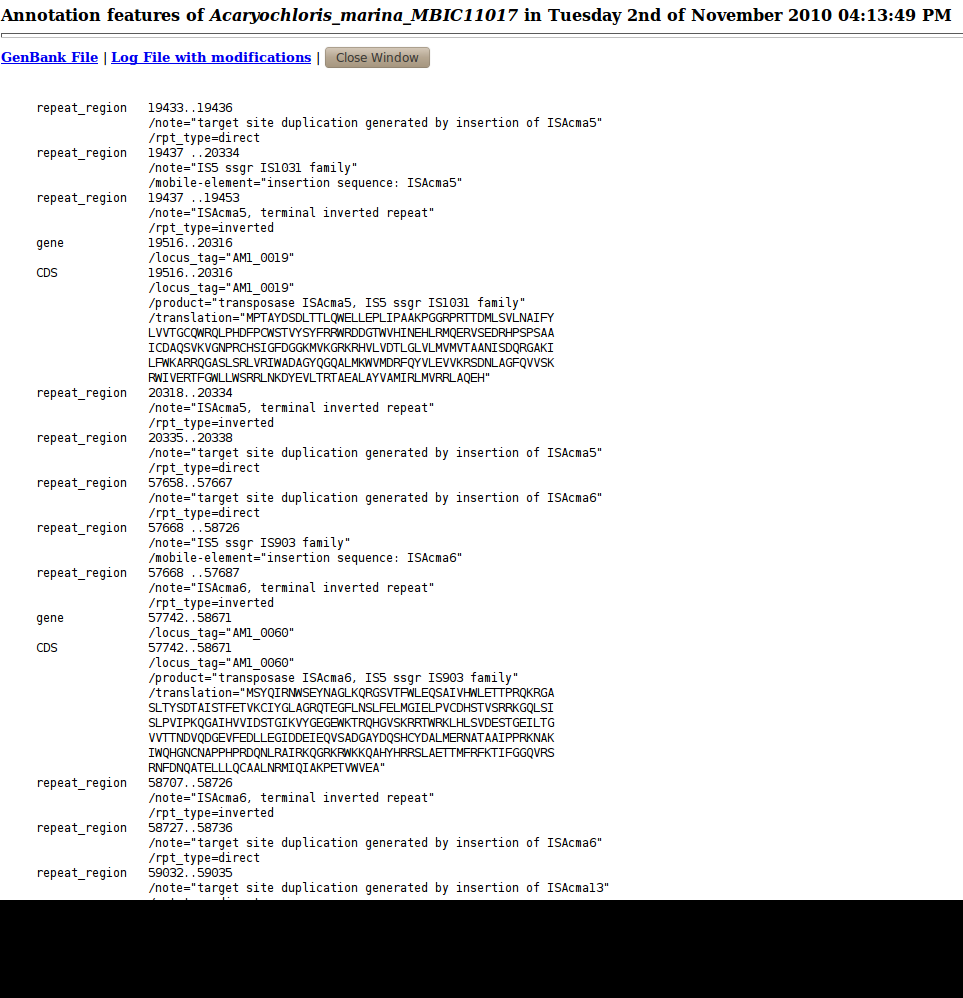


**Figure 6.1.** Example of GenBank annotation extraction.

**Chapter 7. Annotation examples**

ISsaga provides two annotation examples with no account requirements. The first example shows a "work in progress" annotation of the *Acaryochloris marina* MBIC11017 main chromosome. In this example all features for **Annotation of single replicons** in the annotation page menu are active (except the ***Finish the Annotation***) but modifications cannot be saved.

The second example shows a finished and correct IS annotation of the *Stenotrophomonas maltophilia* K279a main chromosome. In this example the **IS Validation Reports** and the **Annotation Table** are open for "read only", and the annotation page menus: ***Predictor & Flanking Region Extractor*** and ***Submit Reference IS copy*** are disabled. The guest user can use this example to familiarise themselves with a correctly finished annotation validated by ISfinder experts.

**
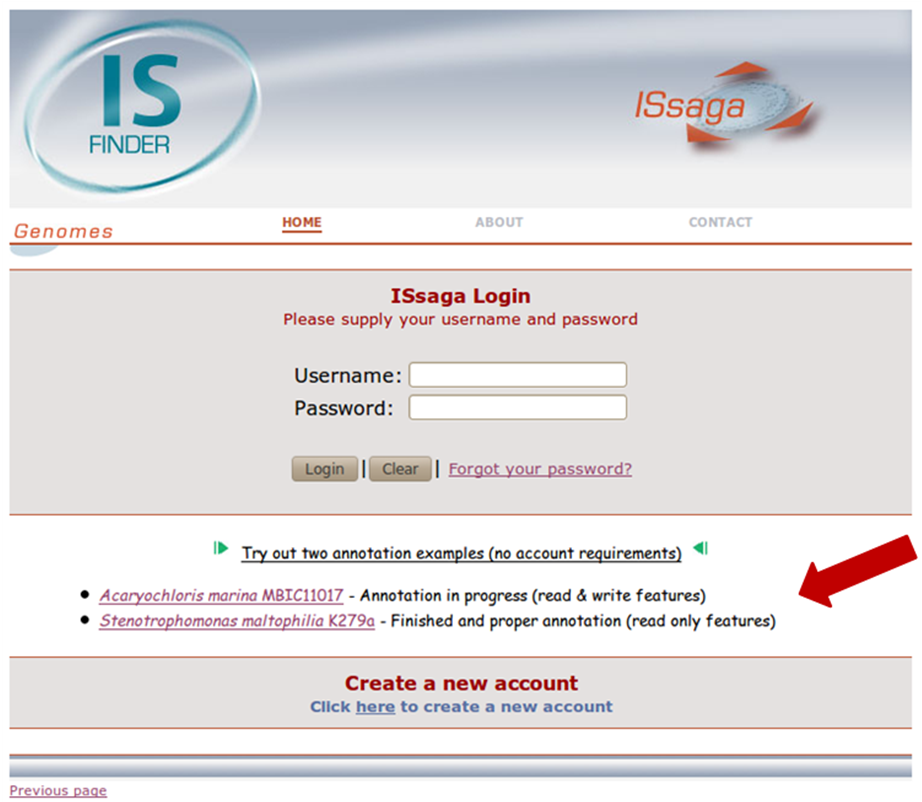
**

**Figure 7.1.** Accessing the annotation examples (red arrow).

**9. Credits**

ISsaga annotation tool was conceived and designed by Alessandro M. Varani, with the help of Patricia Siguier, Edith Gourbeyre and Mike Chandler. The graphics and web-design was provided by David Villa.

The annotation database was created using data provided by members of the Mobile Genetic Elements team (Laboratoire de Microbiologie et Génétique Moléculaire, CNRS, UMR5100,Université Paul Sabatier, Toulouse, FRANCE).

**ISsaga is powered by:**

**BioPerl** - Collection of Perl modules that facilitate the development of Perl scripts for bioinformatics applications.

**BLAST** - Basic Local Alignment Search Tool.

**EMBOSS** - The European Molecular Biology Open Software Suite.

**MySQL** - Relational database management system (RDBMS).

**phpMyEdit** - Instant MySQL Table Editor and PHP Code Generator.

**Sequence Manipulation Suite** - Collection of JavaScript programs for generating, formatting, and analyzing short DNA and protein sequences.

**CGView** - Java package for generating high quality, zoomable maps of circular genomes.

**AmCharts** - Fully customizable animated, 3D or 2D flash charts.

**Jalview** - Multiple alignment editor.

**Readseq** - Read & reformat biosequences.

**ISfinder and ISsaga team**
Alessandro Varani ISsaga Development

Patricia Siguier ISfinder database curation and management

Edith Gourbeyre ISfinder database Curation

Jocelyne Perochon System support

David Villa Graphics

Mike Chandler ISfinder Coordinator

**Support**

| The ISsaga project was supported by the C.N.R.S (Centre National de la Recherche Scientifique) intramural programme. A.V. was supported by The Capes Foundation, Ministry of Education of Brazil. (Grant Number: 2497-08-5) and IBiSA (Infrastructures Biologie Santé et Agronomie) | | |
| --- | --- | --- |
| [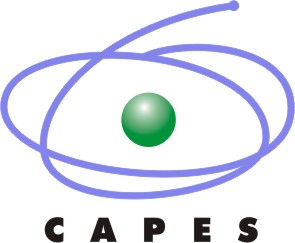](http://www.capes.gov.br/) | 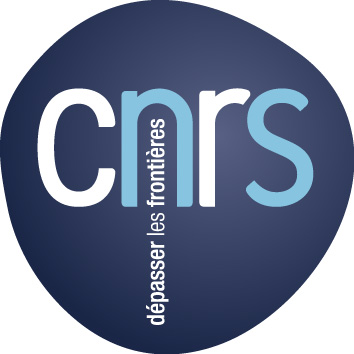 | **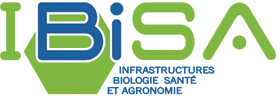** |
