# Supplementary material for: ISsaga is an ensemble of web-based methods for high throughput identification and semi-automatic annotation of insertion sequences in prokaryotic genomes
Source: Genome Biol. 2011 Mar 28;12(3):R30. doi: 10.1186/gb-2011-12-3-r30 (PMC3129680; doi:10.1186/gb-2011-12-3-r30)
Supplement: Additional file 2 — Figure S1 - annotation table. This shows a partially completed annotation table of Acaryochloris marina with its different fields necessary for a proper annotation. The boxes are automatically filled following validation of the ISs in the individual IS reports. Each field is clickable and editable. [file gb-2011-12-3-r30-S2.PDF]

IS FINDER

Replicon Annotation Table

Acaryochloris_marina_MBIC11017 - IS_720150

ISsaga User: **ISsaga guest**

Reload Table

Add IS Page: 1 of 1 Records: 358

Search	IS Name	ORF Name	% of DNA Similarity	% of Similarity AAs *(ISfinder First hit)*	% of AAs Similarity *(Replicon First hit)*	Identified IS Family	ORF Left End	ORF Right End	ORF Size *(bp)*	ORF Size *(aa)*	Strand	Type	IS Left End	IS Right End	IS Length *(bp)*	DR Size *(bp)*	IR Size *(bp)*	IS Coordinates Present *(for partials)*	Length of Partial *(bp)*	General Comments
	ISAcma5	AM1_0019	Reference_copy	100% ISAcma5	100% AM1_0081	IS5 ssgr IS1031	19516	20316	801	266	+	T	19437	20334	898	3	17			
	ISAcma6	AM1_0060	Reference_copy	100% ISAcma6	100% AM1_3283	IS5 ssgr IS903	57742	58671	930	309	+	T	57668	58726	1059	9	18/20			
	ISAcma13	AM1_0061	Reference_copy	100% ISAcma13	100% AM1_2476	IS3 ssgr IS150	59103	59408	306	101	+	T	59036	60298	1263	3	22/25			
	ISAcma13	AM1_0062	Reference_copy	100% ISAcma13	100% AM1_2477	IS3 ssgr IS150	59300	60259	960	319	+	T	59036	60298	1263	3	22/25			
	ISAcma7	AM1_0064	Reference_copy	100% ISAcma7	100% AM1_4078	IS256	62291	61041	1251	416	-	T	62351	61005	1347	7	24/28			
	ISAcma5	AM1_0081	99.55% ISAcma5	100% ISAcma5	100% AM1_0019	IS5 ssgr IS1031	86654	87454	801	266	+	T	86575	87472	898	3	17			

Part of this table has been deleted for simplicity

Search	IS Name	ORF Name	% of DNA Similarity	% of Similarity AAs *(ISfinder First hit)*	% of AAs Similarity *(Replicon First hit)*	Identified IS Family	ORF Left End	ORF Right End	ORF Size *(bp)*	ORF Size *(aa)*	Strand	Type	IS Left End	IS Right End	IS Length *(bp)*	DR Size *(bp)*	IR Size *(bp)*	IS Coordinates Present *(for partials)*	Length of Partial *(bp)*	General Comments
		AM1_6276		100% ISAcma40_aa1	83.33% AM1_1543	IS1	6348789	6348577	213	70	-									
		AM1_6278		85.86% ISAzo20_aa1	76.08% AM1_5416	IS200/IS605 ssgr IS200	6350152	6349865	288	95	-									
	ISAcma16-p	*AM1_6285*	*100% ISAcma16*	*99.65% ISAcma16*	*100% AM1_5567*	*IS4 ssgr IS4*	*6360270*	*6359404*	*867*	*288*	-	*T*		*6359381*				*6360272 (592) - 6359381 (1483)*	*892*	*| Partial Transposase: [182 to 472 - 291 aas] |*
	ISAcma15	AM1_6286	Reference_copy	100% ISAcma15	97.00% AM1_2664	IS630	6361282	6360278	1005	334	-	T	6361355	6360273	1083	2	22/29			
	ISAcma16-p	*AM1_6287*	*99.83% ISAcma16*	*100% ISAcma16*	*100% AM1_6242*	*IS4 ssgr IS4*	*6361907*	*6361347*	*561*	*186*	-	*T*	*6361948*					*6361948 (1) - 6361356 (593)*	*593*	*| Partial Transposase: [1 to 184 - 184 aas] |*
		AM1_6303		94.59% ISAcma37_aa1	94.59% AM1_5283	IS701	6373195	6373308	114	37	+									
		AM1_6304		97.82% ISAcma37_aa1	100% AM1_5503	IS701	6373315	6373455	141	46	+									
	ISAcma8-p		*100% ISAcma8*			*IS701*						*ND*		*6373487*				*6373443 (1352) - 6373487 (1396)*	*45*	*Putative Solo IR*
		AM1_6307		72.30% ISH7A_aa2	48.95% AM1_1947	ISNCY	6377518	6376868	651	216	-									
	ISAcma5	AM1_6309	96.43% ISAcma5	97.74% ISAcma5	100% AM1_6256	IS5 ssgr IS1031	6379093	6378293	801	266	-	T	6379172	6378275	898	3				
	ISAcma6	AM1_6326	99.90% ISAcma6	100% ISAcma6	100% AM1_3283	IS5 ssgr IS903	6395472	6394543	930	309	-	T	6395546	6394488	1059	9	18/20			
	ISAcma27	AM1_6393	97.30% ISAcma27	98.52% ISAcma27	98.52% AM1_0846	IS630	6483088	6483498	411	136	+	T	6483011	6484197	1187	2	17/19			
	ISAcma27-p			*95.18% ISAcma27*			*6483495*	*6483995*	*501*	*166*	+									*| non-annotated partial ORF |*
	ISAcma27	AM1_6394	97.30% ISAcma27	100% ISAcma27	94.96% AM1_0845	IS630	6483576	6484103	528	175	+	T	6483011	6484197	1187	2	17/19			| Partial Transposase: [168 to 183 - 16 aas] |

Figure S1
